# Integration of Morphological Data into Molecular Phylogenetic Analysis: Toward the Identikit of the Stylasterid Ancestor

**DOI:** 10.1371/journal.pone.0161423

**Published:** 2016-08-18

**Authors:** Stefania Puce, Daniela Pica, Stefano Schiaparelli, Enrico Negrisolo

**Affiliations:** 1 Dipartimento di Scienze della Vita e dell’Ambiente, Università Politecnica delle Marche, Ancona, Italy; 2 Dipartimento di Scienze della Terra, dell'Ambiente e della Vita, Università di Genova, Genova, Italy; 3 Museo Nazionale dell'Antartide (MNA, Sede di Genova), Genova, Italy; 4 Dipartimento di Biomedicina Comparata e Alimentazione, Agripolis, Università di Padova, Legnaro, Padova, Italy; Laboratoire de Biologie du Développement de Villefranche-sur-Mer, FRANCE

## Abstract

Stylasteridae is a hydroid family including 29 worldwide-distributed genera, all provided with a calcareous skeleton. They are abundant in shallow and deep waters and represent an important component of marine communities. In the present paper, we studied the evolution of ten morphological characters, currently used in stylasterid taxonomy, using a phylogenetic approach. Our results indicate that stylasterid morphology is highly plastic and that many events of independent evolution and reversion have occurred. Our analysis also allows sketching a possible identikit of the stylasterid ancestor. It had calcareous skeleton, reticulate-granular coenosteal texture, polyps randomly arranged, gastrostyle, and dactylopore spines, while lacking a gastropore lip and dactylostyles. If the ancestor had single or double/multiple chambered gastropore tube is uncertain. These data suggest that the ancestor was similar to the extant genera *Cyclohelia* and *Stellapora*. Our investigation is the first attempt to integrate molecular and morphological information to clarify the stylasterid evolutionary scenario and represents the first step to infer the stylasterid ancestor morphology.

## Introduction

**Fig 1:**
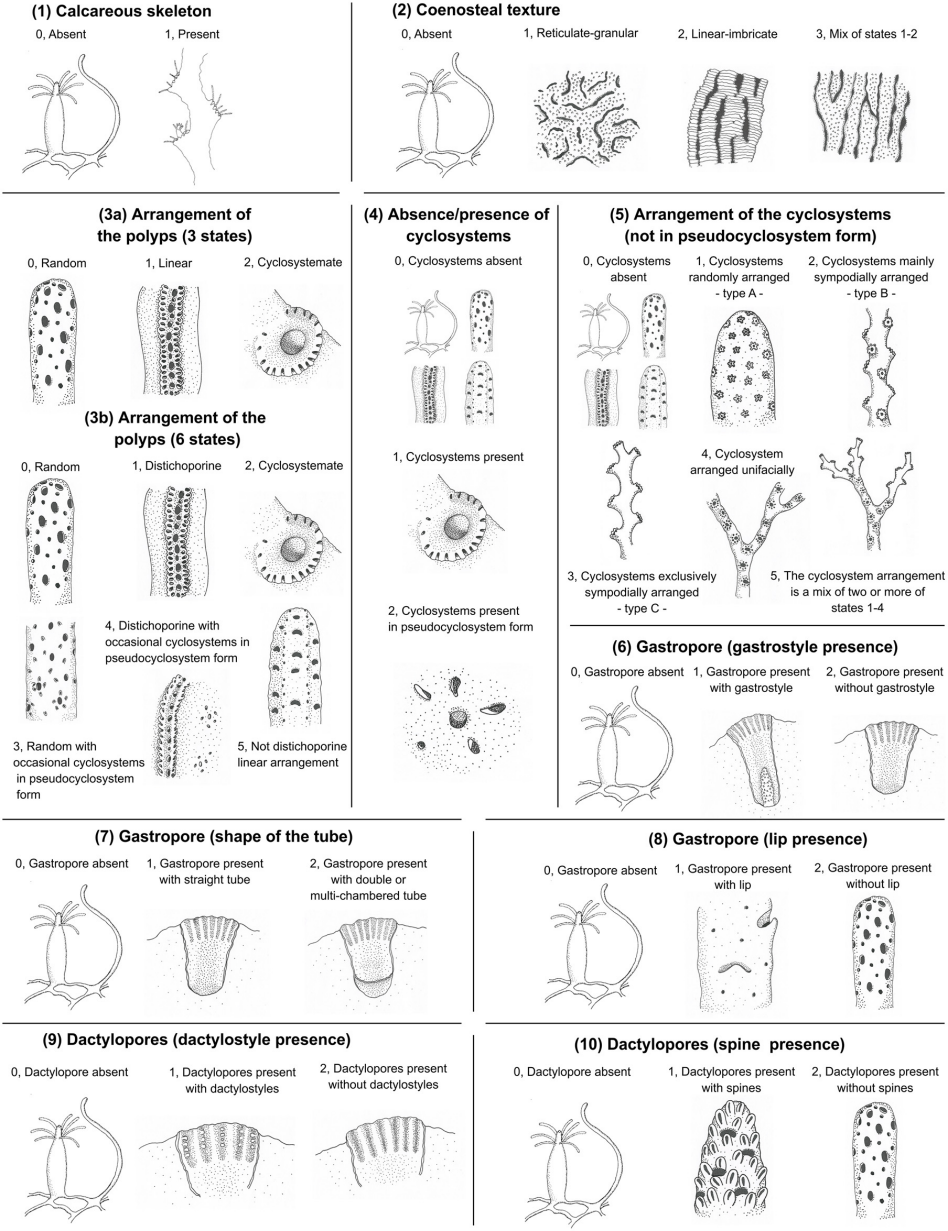
Codes and miniatures depicting the states of the ten characters analysed in present paper.

The Stylasteridae is one of the most speciose hydroid families and includes 29 genera worldwide distributed [1, 2]. The tropical southwest Pacific has been identified as the most diverse region for this family [1, 3]. Stylasterids are characterised by a calcareous skeleton named coenosteum and polymorphic colonies with gastrozooids and dactylozooids arising from pores named gastropores and dactylopores, respectively [4] (see miniatures in Fig 1). The gonophores are placed inside skeletal encasements named ampullae [1, 4]. The gastro- and dactylopores (and consequently the gastro- and dactylozooids) may be irregularly distributed or orderly arranged over the colony. Indeed, eighteen genera are characterised by gastropores and dactylopores randomly arranged along the colony, nine show the gastrozooid surrounded by a circle of several dactylozooids (this arrangement is named cyclosystem) and two usually present one row of gastropores flanked on both sides by a row of dactylopores (it is named distichoporine arrangement) [1, 4] (see miniatures in Fig 1). These features are used as taxonomic characters together with other morphological traits, some of which are usually investigated using scanning electron microscopy. For instance, the arrangement of cyclosystems along the colony, the coenosteal texture, the presence and shape of gastrostyle, the shape of the gastropore tube, the presence of gastropore lip, the presence and shape of dactylostyles and dactylopore spines (see miniatures in Fig 1 and [1] for a complete glossary).

Since long time the evolution of this family stimulates the interest of researchers. In fact, in 1881 Moseley [5] already hypothesised a “pedigree of the Hydrocorallinae” and designed the genus *Sporadopora* as the most ancestral stylasterid. Nevertheless, the first phylogenetic analysis of Stylasteridae was published in 1984 [6]. It was based on a set of 19 morphological characters and *Lepidopora* resulted as the genus with the least number of derived characters. Moreover, the Author reported a relatively high number of homoplasy in the character state changes. The outcomes of a second analysis [7] suggested that *Sporadopora*, *Distichopora* and *Lepidopora* are the least derived genera and that stylasterids progressively evolved from an uncalcified colony with randomly arranged polyps to a well-coordinated “functional unit”.

In 2008 [8] the first molecular phylogenetic analysis, based on mitochondrial 16S ribosomal DNA (rDNA) and nuclear Calmodulin (CaM) and 18S ribosomal DNA (rDNA) markers demonstrated that stylasterid corals originated and diversified in the deep sea. More recently a second molecular phylogenetic analysis was carried out by Lindner et al. [2], to investigate the placement of the new genus and species *Leptohelia flexibilis*. In the same work the new combination *Leptohelia microstylus* was proposed for *Lepidopora microstylus*. From these two analyses [2, 8] three main clades can be identified, one including the five genera *Leptohelia*, *Pliobothrus*, *Conopora*, *Crypthelia* and *Pseudocrypthelia*, a second containing the four genera *Stylaster*, *Calyptopora*, *Stenohelia* and *Stylantheca*, and a third, encompassing the fourteen genera *Stylaster*, *Calyptopora*, *Lepidopora*, *Adelopora*, *Systemapora*, *Stellapora*, *Lepidotheca*, *Errina*, *Errinopsis*, *Stephanohelia*, *Errinopora*, *Inferiolabiata*, *Distichopora* and *Cyclohelia*. Cairns [1], discussing the classification and phylogeny of the family, observed that all three clades may have had an ancestor with polyps arranged in cyclosystems, contrary to the evolutionary trend hypothesised before on morphological characters only [7].

The aim of this work is to investigate the evolution of ten morphological characters by mapping them on a new reference tree obtained through a total evidence approach that combines morphological and molecular characters, in order to sketch an identikit of the stylasterid ancestor.

## Materials and Methods

### Taxa selection

The set of species studied in the present paper was obtained from the data set used recently by Lindner et al. [2], that covers 105 taxa (102 stylasterids and 3 outgroups). From this initial set, we removed the unidentified taxa, i.e. those listed as “cf.”, whose identity and hence morphological attributes were not clear, in order to avoid any possible background noise due to uncertainty in interpretation of the characters. However, the species listed as “cf.” or unidentified in Lindner et al. [2], that were recently identified by Cairns [3], were included in the present work. The final list of taxa used in the present analysis is reported in the Supporting Information (S1 Table).

To facilitate the reading of the text all genera and species are reported using the binomial nomenclature while we provide all taxonomic information and related references in the Supporting Information (S2 Table).

Names used for the species are in agreement with Lindner et al. [2] and Cairns [3]. Moreover, we took into consideration the synonyms proposed by Cairns and Lindner [9] and some changes in the species identifications reported in Cairns [3] that the Author personally communicate us. In Lindner et al. [2] *Lepidotheca macropora* was erroneously listed as *Lepidopora macropora*. Therefore, we replaced the wrong name with the correct one both in figures and tables. Moreover, in accord with Lindner et al. [2] and Cairns [3] we replaced the name *Lepidopora microstylus* with the new combination *Leptohelia microstylus* both in figures and tables.

### Creation of data sets for phylogenetic purposes

The multiple alignment used recently by Lindner et al. [2] for describing the new genus and species *Leptohelia flexibilis*, represents the most complete molecular data set available for Stylasteridae and covers 105 taxa, outgroups included (hereafter listed DNA.105T set). The DNA.105T set is 2638 positions long and was very kindly provided by Dr. Lindner. The DNA.105T was produced by concatenating portions of the mitochondrial 16S and of the nuclear 18S and calmodulin genes [2]. Starting from DNA.105T, we removed all the sequences that were associated to not fully identified taxa i.e. those listed with “cf.” (see above for the rationale of this choice). Furthermore, when multiple specimens were present for the same species (e.g. *L*. *flexibilis*), only one was retained to allow a balanced treatment of the taxonomic diversity. The only exceptions were the specimens attributed to the same species on a morphological ground [3] that do not group together in the reference tree provided by Lindner et al. [2]. After this removing activity, a data set containing 92 taxa remained (listed hereafter as DNA.92T). A second set, including the coded morphological characters described below and encompassing the same species of DNA.92T, was also created (listed hereafter as MRP.92T). DNA.92T and MRP.92T were concatenated in a unique data set (TOT.92T) including both molecular and morphological characters. DNA.92T and TOT.92T sets were used in the phylogenetic analyses as detailed in the next paragraph.

A thorough inspection of DNA.105T set revealed that several taxa were represented in the alignment by a single/two genes while the remaining marker/s were missing and coded as indels. The same reasoning applied also to DNA.92T set (see S3 Table). This finding lead us to treat the DNA set as a single partition.

### Phylogenetic analyses

An *a priori* estimation of the conflicting phylogenetic signals [10] existing in the DNA.105T, DNA.92T and MRP.92T sets was performed by computing neighbor-nets with the SplitsTree program [11] (see S1–S3 Figs).

Phylogenetic trees were inferred using Bayesian inference (BI) and maximum likelihood (ML) methods [12]. The BI trees were obtained with MrBayes 3.2.6 [13]. Two simultaneous runs, each of four chains, were performed in all analyses. Each run consisted of 30,000,000 generations, and trees were sampled every 500 generations (trees generated = 6 x 10^4^). Stationarity was considered to be reached when the average standard deviation of split frequencies was less than 0.005. Burn-in was very stringent and only the last 2,000 generated trees were used to compute the majority-rule posterior consensus trees. The ML trees were computed with the program IQ-TREE 1.4.2 [14], which allows the simultaneous analysis of both morphological and molecular characters. In each analysis 100 independent searches were performed in order to avoid/minimize the possibility to be entrapped in sub-optimal trees. The evolutionary model applied to molecular data sets in both Bayesian and Likelihood models was the GTR+I+G [15], while the MK model of Lewis was used for the morphological characters [16].

### Statistical tests on tree topology

The ultrafast bootstrap (UFBoot) tests [17] were performed to assess the robustness of ML tree topologies (10,000 replicates in all cases). Alternative topologies were evaluated using the weighted Shimodaira and Hasegawa (WSH) [18] and the approximately unbiased (AU) test [18] implemented in the IQ-TREE program [14]. The compared topologies were the ML trees obtained from DNA.92T and TOT.92T sets and a third tree (named hereafter as LIN tree) obtained from the ML tree originally published by Lindner et al. [2], by removing the branches connecting taxa non included in DNA.92T and TOT.92T sets.

### Description of morphological characters

The evolution of ten morphological characters was studied in the present work. The considered characters and their states are described below (see Fig 1 for more details and S1 Table for the morphological matrix).

(1) Calcareous skeleton. Two are the states for this character: absent, 0; present, 1.
(2) Coenosteal texture. It is a four-states character: absent, 0; reticulate-granular, 1; linear-imbricate, 2; mix of states 1–2, 3.
(3) Arrangement of the polyps. This feature was coded in two different ways. In a first approach (3a), three states were used to describe the arrangement of the polyps. These three states represent the morphological categories typically used in the species descriptions obtained from literature. In a second more detailed approach (3b) the polyp arrangement was considered a six-states character including also the occasional presence of cyclosystems in pseudocyclosystem form and a linear arrangement different from the distichoporine one.
(3a) Arrangement of the polyps (3 states): random, 0; linear, 1; cyclosystemate, 2.
(3b) Arrangement of the polyps (6 states): random, 0; distichoporine, 1; cyclosystemate, 2; random with occasional cyclosystems in pseudocyclosystem form, 3; distichoporine with occasional cyclosystems in pseudocyclosystem form, 4; not distichoporine linear arrangement, 5.
(4) Absence/presence of cyclosystems: cyclosystems absent, 0; cyclosystems present, 1; cyclosystems present in pseudocyclosystem form, 2.
(5) Arrangement of the cyclosystems (not in pseudocyclosystem form): cyclosystems absent, 0; cyclosystems randomly arranged -type A-, 1; cyclosystems mainly sympodially arranged -type B-, 2; cyclosystems exclusively sympodially arranged -type C-, 3; cyclosystem arranged unifacially, 4; cyclosystem arrangement that is a mix of two or more of the states 1–4, 5.
(6) Gastropore (gastrostyle presence): gastropore absent, 0; gastropore present with gastrostyle, 1; gastropore present without gastrostyle, 2.
(7) Gastropore (shape of the tube): gastropore absent, 0; gastropore present with straight tube, 1; gastropore present with double or multi-chambered tube, 2.
(8) Gastropore (lip presence): gastropore absent, 0; gastropore present with lip, 1; gastropore present without lip, 2.
(9) Dactylopores (dactylostyle presence): dactylopores absent, 0; dactylopores present with dactylostyles, 1; dactylopore present without dactylostyles, 2.
(10) Dactylopores (spine presence): dactylopores absent, 0; dactylopores present with spines, 1; dactylopores present without spines, 2.

The states of the characters (S1 Table) were obtained from taxa descriptions present in the literature [3, 4, 9, 19–27]. In the case of *Distichopora irregularis* we studied the features directly on the holotype (BMNH 80.11.25.173) deposited in the collections of Natural History Museum of London (UK).

The characters and their states were defined following the glossary of Cairns [1] with the exception of the cyclosystem and the dactylopore spines. He defined the cyclosystem as “a functional unit of stylasterid colony structure composed of a central gastropore (gastrozooid) surrounded by a variable number of dactylopores (dactylozooids)”. In the revision of Alaskan stylasterids, Cairns and Lindner [9] defined the pseudocyclosystem as “a cyclosystem-like structure (composed of a gastropore surrounded by dactylopores) that may be found at basal branches of some stylasterid species and, in which, the dactylopores are usually not fused with the central gastropore and may not have the slits (dactylotome) of the dactylopore spines directed towards the central gastropore”. The partial overlapping of the two definitions and the use of terms as “usually” or “may” suggests that it is very hard to clearly distinguish these structures. The unique stable difference is that cyclosystems occur in cyclosystemate species, while pseudocyclosystems occur in basal branches of non-cyclosystemate species. Therefore, we have considered the pseudocyclosystems as a form of cyclosystems that occasionally may occur in non-cyclosystemate species.

Moreover, differently from the definition provided by Cairns [1], we have considered as having spines all dactylopores elevated from the coenosteal surface or with any projection from the coenosteum, regardless of its shape.

### Tracking the evolutionary pathways of characters

The evolution of the ten characters described above was studied by mapping their transformation pathways along the ML trees obtained from the phylogenetic analyses performed on DNA.92T and TOT.92T (see below Results). In these trees the nodes receiving BT support lower than 50% were collapsed and the resulting cladograms were used as the reference trees to map the evolution of morphological traits. The characters were studied according to the maximum likelihood approach by applying the MK1 model of Lewis [16]. The analyses were done using the software Mesquite [28].

## Results

### Reconstruction of phylogenetic trees

The neighbor-nets produced with SplitsTree program reveal that conflicting phylogenetic signals exist in DNA.105T, DNA.92T and MRP.92T sets (S1–S3 Figs). In the case of DNA.105T and DNA.92T sets the missing genes probably play a pivotal role in this behaviour (see above Materials and methods). It was not possible to analyse TOT.92T with SplitsTree, because the current version of the program does not handle mixed types of characters.

**Fig 2:**
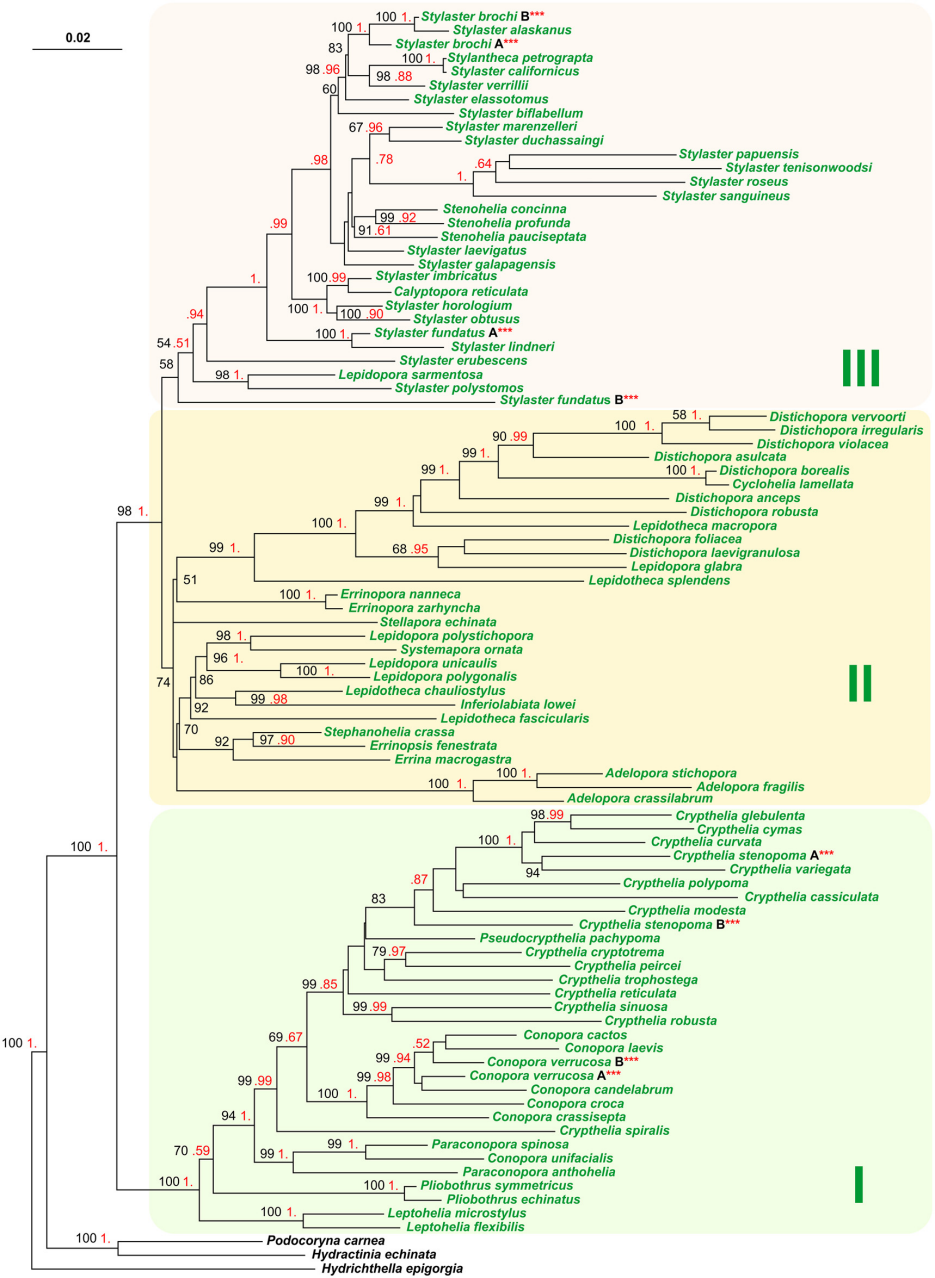
Maximum likelihood tree obtained from the analysis of TOT.92T data set. The ML tree (-ln = 23933.8193) was computed with IQ-TREE program. The scale bar represents 0.02 substitutions/state change per position. Black numbers represent ultrafast bootstrap values (>50%) expressed in percent, while red numbers refer to Bayesian Inference posterior probabilities. These latter values are provided in a compressed way (e.g. 1. instead of 1.00; .95 instead of 0.95) to allow a better readability of the figure.

The ML phylogenetic tree obtained from TOT.92T is provided in Fig 2. Several nodes receive BI/UFBoot support. Three main clades (I to III) can be identified in the topology. Clade I is sister taxon of the II + III clades, and these relationships receive BI/ UFBoot corroborations. Several genera appear para-polyphyletic (e.g. *Lepidotheca*, *Lepidopora*, *Stylaster*). In the phylogenetic tree obtained from DNA.92T (S4 Fig) the clades I-III are also present. Taxa composition for the three mains clades is the same observed for TOT.92T tree with the exceptions represented by the placement of *Stylaster erubescens*, *Stylaster fundatus*, *Stylaster polystomos* and *Lepidopora sarmentosa* in clade II instead of clade III (Fig 2; S4 Fig). Conversely, phylogenetic relationships within each major clade are not congruent. Finally, some specimens that are indistinguishable on a morphological point of view and considered members of the same species, result to be distinct taxa when the DNA is considered (e.g. *Crypthelia stenopoma* A and *C*. *stenopoma* B) (Fig 2; S4 and S5 Figs).

The alternative topologies tests performed on TOT.92T tree (Fig 2), DNA.92T tree (S4 Fig) and LIN tree (S5 Fig) show a compatibility among the three topologies in the calculation performed on TOT.92.set (p-WSH >0.15, p-AU > 0.14). Conversely, the tests on DNA.92T.set identify a mild incompatibility among TOT.92T tree and the two trees obtained from molecular data only (p-WSH = 0.05; p-AU = 0.04). The second and third trees are fully compatible (p-WSH = 0.43; p-AU = 0.31). Finally, the DNA.92T tree exhibits a better likelihood value that LIN tree (-23439.825 vs. -23446.722). All these results lead us to use the TOT.92T tree (Fig 2) as reference phylogenetic tree to track the evolution of morphological characters.

### Evolution of morphological traits in Stylasteridae

**Fig 3:**
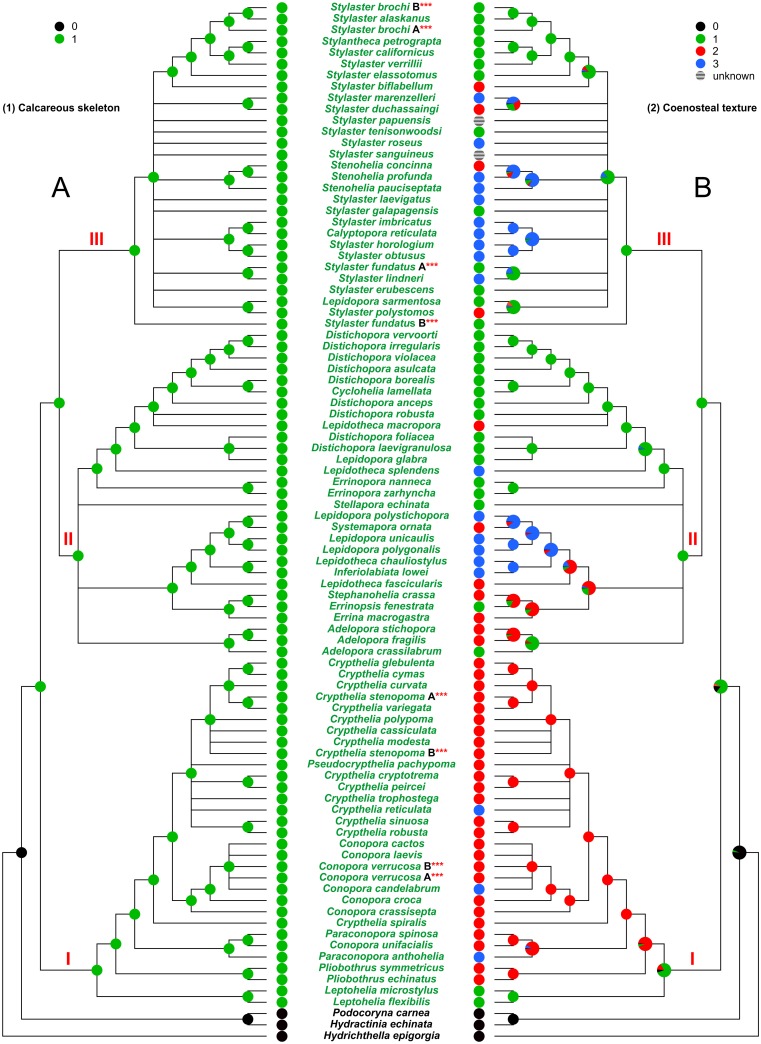
Evolution of the characters 1 and 2. (A) Character 1, calcareous skeleton. (B) Character 2, coenosteal texture. I, II, and III, major clades cited in the text. The state of the analysed character is represented by a coloured pie, placed at each internal/terminal node of the tree. An enlarged multi-coloured pie is used when multiple states of a character occur at a specific node. In this latter case the size of each slice is proportional to the probability of occurrence of the state.

**Fig 4:**
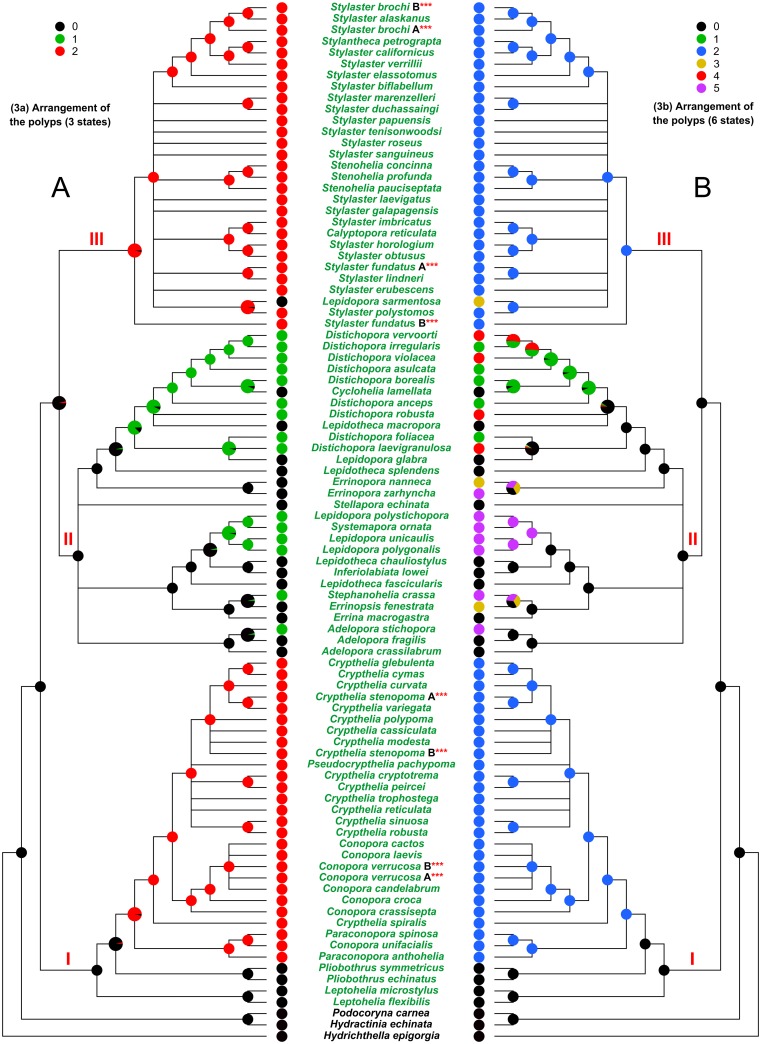
Evolution of the characters 3a and 3b. (A) Character 3a, arrangement of the polyps (three states). (B) Character 3b, arrangement of the polyps (six states). I, II, and III, major clades cited in the text. The state of the analysed character is represented by a coloured pie, placed at each internal/terminal node of the tree. An enlarged multi-coloured pie is used when multiple states of a character occur at a specific node. In this latter case the size of each slice is proportional to the probability of occurrence of the state.

**Fig 5:**
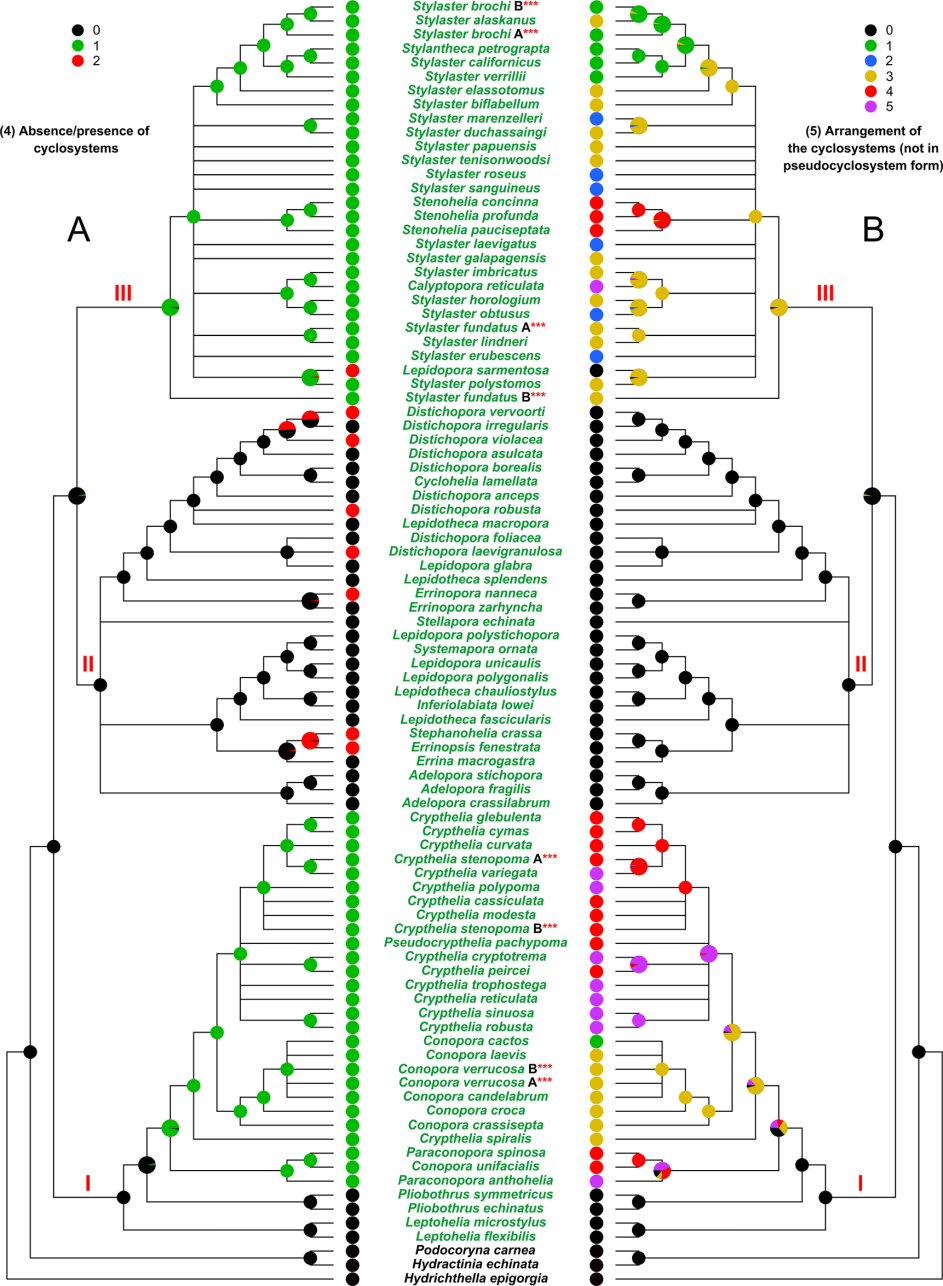
Evolution of the characters 4 and 5. (A) Character 4, absence/presence of cyclosystems. (B) Character 5, arrangement of the cyclosystems (not in pseudocyclosystem form). I, II, and III, major clades cited in the text. The state of the analysed character is represented by a coloured pie, placed at each internal/terminal node of the tree. An enlarged multi-coloured pie is used when multiple states of a character occur at a specific node. In this latter case the size of each slice is proportional to the probability of occurrence of the state.

**Fig 6:**
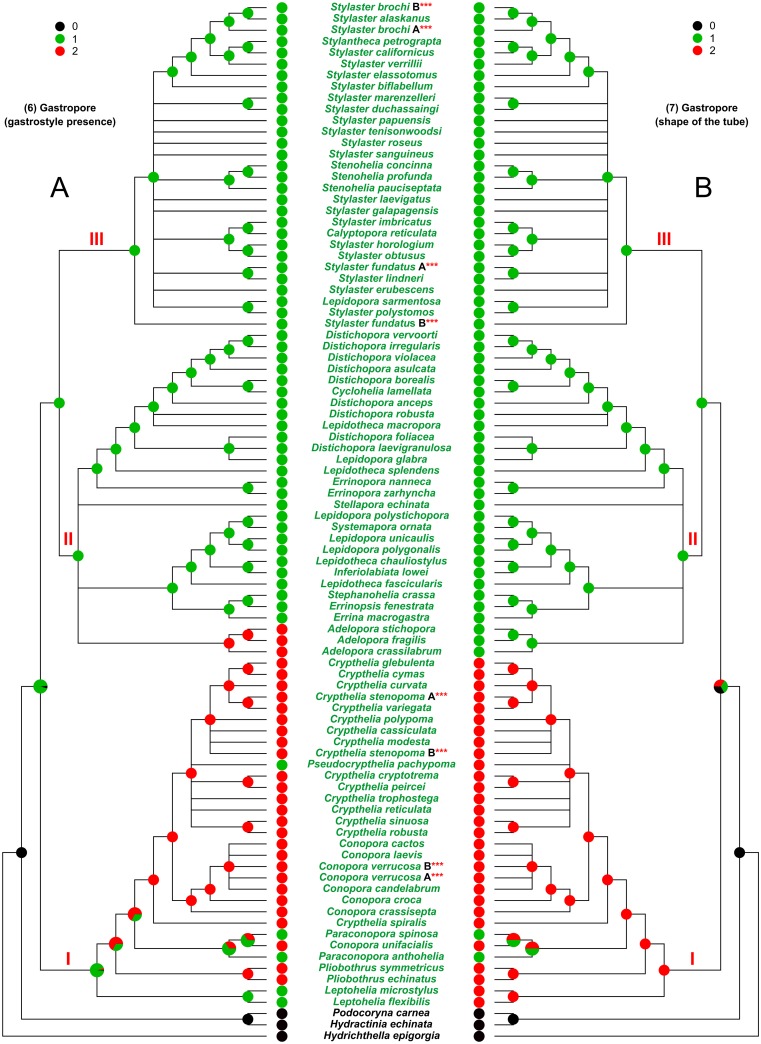
Evolution of the characters 6 and 7. (A) Character 6, gastropore (gastrostyle presence). (B) Character 7, gastropore (shape of the tube). I, II, and III, major clades cited in the text. The state of the analysed character is represented by a coloured pie, placed at each internal/terminal node of the tree. An enlarged multi-coloured pie is used when multiple states of a character occur at a specific node. In this latter case the size of each slice is proportional to the probability of occurrence of the state.

**Fig 7:**
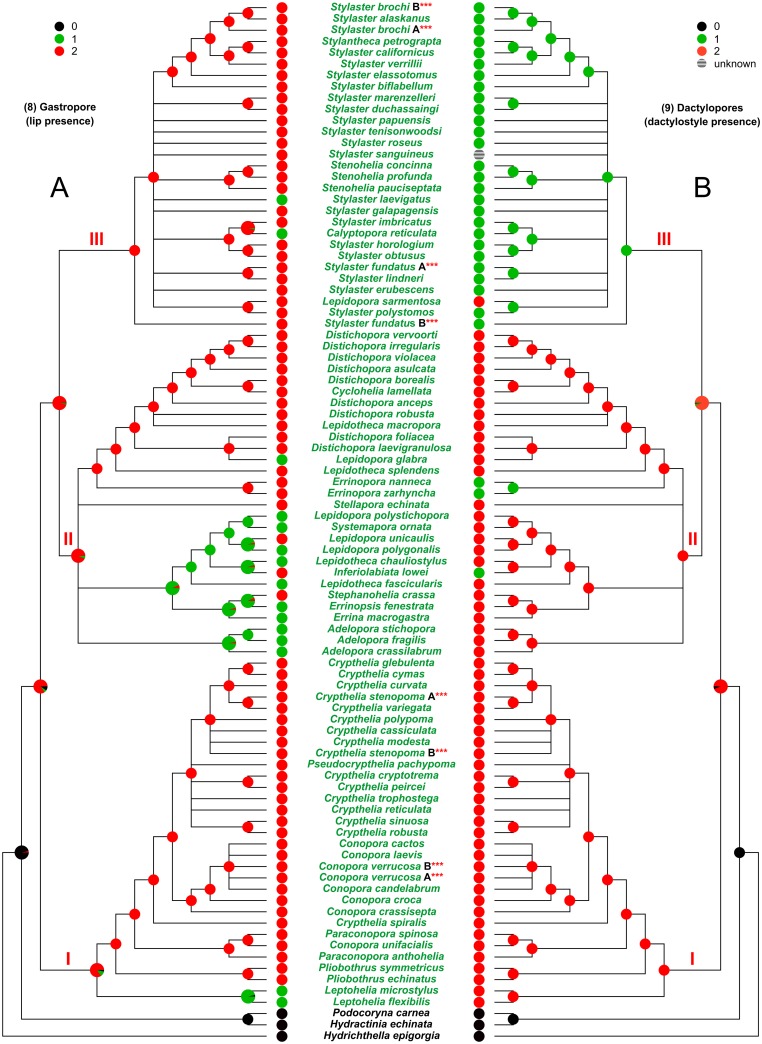
Evolution of the characters 8 and 9. (A) Character 8, gastropore (lip presence). (B) Character 9, dactylopores (dactylostyle presence). I, II, and III, major clades cited in the text. The state of the analysed character is represented by a coloured pie, placed at each internal/terminal node of the tree. An enlarged multi-coloured pie is used when multiple states of a character occur at a specific node. In this latter case the size of each slice is proportional to the probability of occurrence of the state.

**Fig 8:**
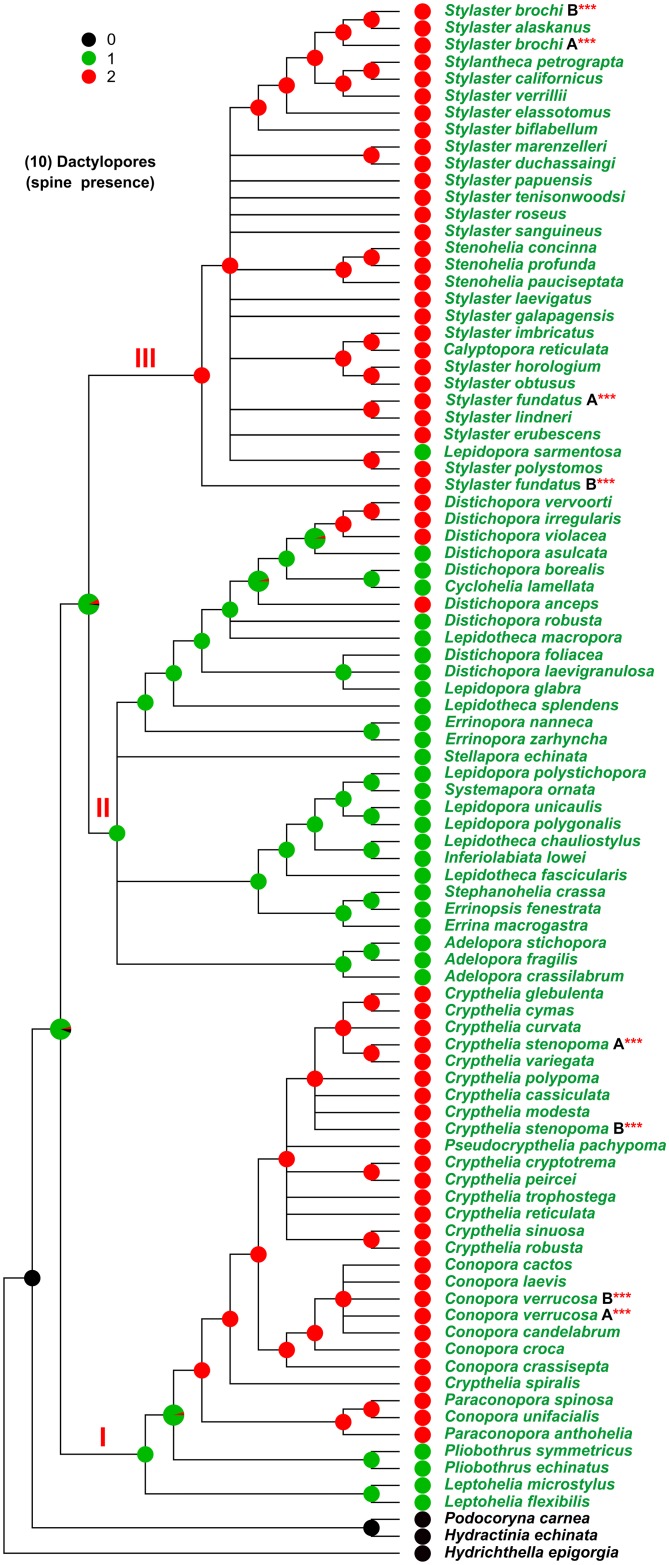
Evolution of the character 10. Character 10, dactylopores (spine presence). I, II, and III, major clades cited in the text. The state of the analysed character is represented by a coloured pie, placed at each internal/terminal node of the tree. An enlarged multi-coloured pie is used when multiple states of a character occur at a specific node. In this latter case the size of each slice is proportional to the probability of occurrence of the state.

The evolution of ten morphological characters (Fig 1; S1 Table) was mapped on cladograms obtained from the DNA.92T and TOT.92T trees by collapsing the nodes having UFBoot support < 50% (see Materials and methods). The results obtained from these analyses are largely congruent, and the overall evolutionary scenarios do not change (see below). Very minor differences exist, due to the diverse placement of some taxa (see section above). Thus, in the main text only the findings relative to the TOT.92T analysis are presented in details (Figs 3–8). Results of the character-mapping, performed on the DNA-based tree, are provided for completeness as supporting material (S6–S11 Figs).

To facilitate the comprehension of changes along the tree (Figs 3–8), the colour corresponding to a state of a character is provided in brackets in the next paragraphs.

A calcareous skeleton (green) (character 1) was acquired by the ancestor of Stylasteridae and is maintained through all its descendants (Fig 3A).

The coenosteal texture (character 2) (Fig 3B) was most probably reticulate-granular (green) in the ancestor and a linear-imbricate texture (red) was independently acquired nine times. This derived condition characterises most of the species included in clade I. The parallel evolution of the mixed texture (blue) occurred in all the main clades.

When the “arrangement of the polyps” is considered as a three states character (character 3a) (Fig 4A) the ancestral state of the family is represented by the random arrangement (black). The appearance of the cyclosystemate arrangement (red) occurred two times: it characterises the whole clade III (with the exception of *Lepidopora sarmentosa*) and a subclade (including the genera *Paraconopora*, *Conopora*, *Crypthelia* and *Pseudocrypthelia*) inside clade I. The linear arrangement (green) is exclusively present in clade II where it was independently acquired four times. Furthermore, at minimum three reversions to the ancestral state occurred (i.e. *L*. *sarmentosa*, *Cyclohelia lamellata* and *Lepidotheca macropora*).

The situation is similar when the “arrangement of the polyps” is treated as six states character (character 3b) (Fig 4B). The random arrangement (black) appears to be the ancestral state and the cyclosystemate arrangement (blue) evolved independently in clade I and in clade III. The other four states of this character are present exclusively in clade II and III. The random arrangement results to be the ancestral state of clade II. The evolution of distichoporine arrangement (green) does not appear clearly resolved. Indeed, this arrangement results to have evolved independently two to three times. The parallel evolution of the arrangement distichoporine with pseudocyclosystems (red) occurred two to four times. Finally, the random with occasional cyclosystems in pseudocyclosystem form (yellow) and not-distichoporine linear (purple) arrangements were acquired independently three and four times respectively.

The transformation pathway of the absence/presence of cyclosystems (character 4) (Fig 5A) shows that the ancestor of the family was characterised by pores not arranged in cyclosystems (black) and that the cyclosystems (green) appeared independently two times. Furthermore, in clade II the evolution of cyclosystems in pseudocyclosystem form (red) happened independently six / seven times.

The analysis of the arrangement of cyclosystems (character 5) (Fig 5B) confirms that in the ancestor of the family the pores were randomly arranged. Moreover, it reveals that the sympodial organization (yellow) (type C) evolved multiple times, the “type B” arrangement (blue) appeared separately at minimum three times and the “type A” (green) was acquired twice. The unifacial arrangement (red) evolved independently four times. Finally, the “mixed” arrangement (purple) appeared separately five times.

The mapping of the gastrostyle presence inside the gastropore (character 6) (Fig 6A) shows that the family ancestor was provided with it (green). However, the gastrostyle was independently lost (red) once in clade II and one to three times in clade I where it successively reappeared in *Pseudocrypthelia pachypoma* and maybe in *Paraconopora spinosa* and *Paraconopora anthohelia*.

The ancestral state of character 7 (shape of the gastropore tube) (Fig 6B) is not resolved in our analysis. The single chambered gastropore tube (red) is present in clades II and III while clade I is characterised by double/multiple chambered gastropore tube except for one to two independent acquisition of single chambered gastropore tube in *P*. *spinosa* and *P*. *anthohelia*.

The analysis of the gastropore lip presence (character 8) (Fig 7A) shows that the absence of the lip (red) was probably the ancestral state. The gastropore lip appeared independently (green) five to six times.

The mapping of the presence of dactylostyles inside the dactylopores (Character 9) (Fig 7B) indicates that they were very probably absent (red) in the family ancestor and that they successively evolved (green) and characterised almost the entire clade III. The dactylostyles were independently acquired twice in the clade II.

Finally, the analysis of the presence of the dactylopore spines (character 10) (Fig 8) reveals that they were present (green) in the family ancestor and were independently lost (red) four times. The absence of dactylopore spines characterises the whole clade III (with the exception of *L*. *sarmentosa*) and a large subclade inside clade I.

## Discussion

Until present paper two molecular phylogenies have been produced for Stylasteridae [2, 8]. Both studies revealed the presence of many para/polyphyletic genera. Furthermore, some genera such as *Stylaster* and *Calyptopora* were even split in different clades. Conversely, very few genera were found to be monophyletic, i.e. *Adelopora*, *Errinopora* and *Pliobothrus*. Lindner et al. in 2008 [8] only partially discussed the taxonomic outputs of their phylogenetic analysis. Conversely, they focused their attention on the bathymetric distribution of the species demonstrating that stylasterids originated and diversified in deep water and invaded several times shallow water. The 2014 phylogeny of Lindner et al. [2] is similar to that published in 2008. However, some taxa changed their placement due also to the addition of specimens belonging to the new genus *Leptohelia*. Lindner et al. [2] discussed the presence of a rudimentary gastrostyle in *Leptohelia* and *Pseudocrypthelia pachypoma* hypothesising that they may have evolved this structure independently.

As described by Cairns [1], the 2008 phylogenetic analysis of Lindner et al. [8] identified three major clades that were confirmed also by the successive analysis performed in 2014 [2]. In present work we identified also three major clades, that however do not match perfectly the composition of previous analyses (Fig 2; S4 and S5 Figs). In our analyses both based on total evidence approach (TOT.92T set) and molecular data (DNA.92T) many genera still are para/polyphyletic (Fig 2; S4 Fig). However, the *Stylaster* genus is no longer split in two main clades and all species are firmly nested within clade III in TOT.92T tree suggesting a positive effect of morphological data in reducing the incongruence among phylogenetic outputs and current taxonomic arrangement. Conversely *Lepidopora sarmentosa* that in the DNA trees (S4 and S5 Figs) was included in the clade II, together with other *Lepidopora* species, has moved to clade III in the TOT.92T. Finally, samples assigned to the same morphological species [3], result to be distinct taxa in the DNA.92T analysis (Fig 2; S4 and S5 Figs). This latter finding suggests that a strong disagreement exist at least for some taxa between the species identification on morphological vs molecular basis.

The split analysis (S1 and S2 Figs) revealed conflicting phylogenetic signals in molecular data that we attribute to the high amount of missing sequences (see Materials and methods and Results sections). The morphological data also show the existence of conflicting signals that were very probably determined by the very limited number of characters (S3 Fig).

Despite these limits, by combining morphological characters with molecular data we were able to obtain a total evidence robust phylogeny to study the evolution of ten morphological characters.

Our study provides new evidence on our understanding on the evolution of Hydrozoa. Previously, the life cycle and colonial organization in leptothecates has been investigated in a phylogenetic framework by Leclère et al. [29, 30]. Furthermore, two analyses have been addressed to the sister group of stylasterids by Miglietta and co-workers. In a first paper, Miglietta et al. [31] studied the evolution of calcareous skeleton in hydractiniids, while Miglietta and Cunningham [32] investigated the evolution of life cycle, morphology of the colony and host specificity in the same family. Very recently, Mendoza-Becerril et al. [33] studied the evolution of the exoskeleton in Medusozoa including in their analysis several anthoathecate taxa such as Stylasteridae and Hydractiniidae.

The present analysis shows that the considered characters exhibit high plasticity and frequent events of independent evolution (Figs 3–8) leading to similar morphologies in not strictly related clades. In his phylogenetic analysis, also Cairns [6] noticed many cases of reversal, parallelism or convergences suggesting that “stylasterids were quite convergent in their evolution” or “the characters chosen to produce the cladogram are not conservative at the generic level”.

The more plastic characters appear to be the arrangement of the polyps (Figs 4A, 4B and 5A), the arrangement of the cyclosystems (Fig 5B) and the coenosteal texture (Fig 3B).

The arrangement of the polyps was mapped considering both three and six states (Fig 4A and 4B). The ancestral state clearly results to be “polyps randomly arranged” in both the analyses. Moreover, the analysis of the presence/absence of cyclosystems (Fig 5A) identifies the non-cyclosystemate arrangement of polyps as the plesiomorphic condition for the family. Cyclosystems, in form of pseudocyclosystems, appeared repeatedly, during the evolution of the group, in seven species belonging to clade II, i.e. *Errinopsis fenestrata*, *Stephanohelia crassa*, *Errinopora nanneca*, *Distichopora laevigranulosa*, *Distichopora robusta*, *Distichopora violacea*, *Distichopora vervoorti* and also in *L*. *sarmentosa* which belongs to clade III (Figs 4B and 5A). Puce et al. [34, 35] have confirmed the production of a primary cyclosystem during the ontogeny of cyclosystemate species (*Stylaster* sp.) and demonstrated that it occurs also in non-cyclosystemate ones (*Distichopora* cf. *violacea* and *Distichopora* cf. *nitida*). Additional observations (present paper) carried out on colonies present in the collections of Natural History Museum of London (UK), Natural History Museum of Leiden (Netherland), Muséum National d'Histoire Naturelle of Paris (France) and Museo Nazionale dell’Antartide Felice Ippolito of Genova (Italy), show that the formation of a primary cyclosystem is very frequent in both the cyclosystemate and non-cyclosystemate genera (*Stylaster*, *Conopora*, *Pliobothrus*, *Errina*, *Lepidotheca*, *Systemapora*, *Stephanohelia*).

Cairns [7] hypothesised an evolutionary trend composed of eleven steps. He placed the evolution of polyps coordinated in cyclosystems at the step seven and stated that this polyp arrangement is characteristic of all the highly derived stylasterid genera. Our analyses regarding the arrangement of polyps (Fig 4A and 4B) and the presence of cyclosystems (Fig 5A) identify the independent onset of cyclosystemate species in two of the three main clades (clades I and III). Moreover, the cyclosystems occur several times in form of pseudocyclosystems in clades II and III. Considering the cyclosystem as a complex structure, the multiple independent evolution of this trait is rather unlikely if it is linked to a tightly regulated genetic control. However, the frequent occurrence of primary cyclosystems indicates that also non-cyclosystemate species are able to produce this arrangement. This latter observation suggests that the cyclosystem could be a structure less complex than previously hypothesised. In addition, the cyclosystem clearly allows trophic efficiency and protection to the gastrozooids of an adult colony [7] and even more so to the first gastrozooid of a new colony. Therefore, the presence of this functional unit in numerous young stylasterids may be related to a shared ecological pressure at the early stages of life of these organisms.

The arrangement of cyclosystems represents a very plastic character (Fig 5B). Furthermore, the reconstruction of state characters for several nodes, especially in clade I, is not fully resolved. Nevertheless, a subclade including nine species with a unifacial arrangement (red dots) and seven species with a mixed arrangement (purple dots) can be recognised in clade I. Actually, the mixed arrangement is an unifacial arrangement with cyclosystems that sometime may also occur on the other face of the colony, therefore the entire subclade might be considered basically unifacial. As already noted by Lindner et al. [8] this subclade is also bearing the innovation of a calcified lid that protects the gastropore. It is also interesting that different colonies of *Stylaster brunneus*, a species not included in the phylogenetic tree, have been described with cyclosystems arranged as a *Stylaster* type A, type B or type C [19]. Cairns [19] noted that the cyclosystem arrangement is probably related to water turbulence. This observation also suggests that this character is very plastic.

Recurrent parallel evolution and reversion events are also observed in the distribution of the different states of the character “coenosteal texture” (Fig 3B). The reticulate-granular texture represents the most probable ancestral state from which the linear-imbricate and mixed textures have been evolved. The complex evolutionary pattern of coenosteal texture may be related to the high variability of this character that sometime occurs in different states when one considers different portions of the same colony as repeatedly reported in taxonomic literature (e.g. [3, 19]).

The results of the present investigation allow sketching a first identikit of the possible stylasterid ancestor and of its morphological characters. It had calcareous skeleton, reticulate-granular coenosteal texture, polyps randomly arranged, gastrostyle, and dactylopore spines while was lacking gastropore lip and dactylostyles. The presence of the calcareous skeleton in the stylasterid ancestor is in accord with the hypothesis of only one event of skeleton evolution in this family as also hypothesised for milleporids [31]. Moreover, Cairns [3] and Mendoza-Becerril et al. [33] suggested that the calcareous skeleton may be one of the characters that led to the evolutionary success of Stylasteridae. Cairns [3] suggested that the gastrostyle not only provides an anchor to the gastrozooid but it represents also a protective structure from predators. Furthermore, he considered also the double-chambered gastropore tube as a morphological structure useful to deter predators. Our analysis indicates that the ancestor had gastrostyle but it is uncertain if it had a single or double/multiple chambered gastropore tube.

The pattern of character states of the ancestor resembles the morphology of the two extant genera *Cyclohelia* and *Stellapora*. These two genera are monotypic and are known from Alaska and sub-Antarctic area, respectively. Moreover, also the sub-Antarctic genera *Cheiloporidion* and *Errinopsis* share with the ancestor the states of all the considered characters with the exception of some coenosteal texture details. These four genera are from geographic areas close to Poles and located inside FAO oceanic regions 17 and 18 [1]. In addition, they are from deep water and include only one or two known species.

Based on the observation that “styles appear in some genera in the dactylopores as well as in the gastropores”, Moseley [5] hypothesised that both gastrostyles and dactylostyles were present in the “Archistylaster”. Moreover, he identified *Sporadopora* as the most ancestral stylasterid assuming that the styles present in the “Archistylaster” disappeared in this genus and reappeared by reversion in *Stylaster* and *Allopora* (currently synonymised with *Stylaster*). The ancestor resulting from our analysis partially agree with Moseley’s hypothesis owing to the absence of dactylostyles. In addition, for the same reason, the presence of dactylostyles in *Stylaster* appears not related to a reversion event. Broch considered *Pliobothrus* [36] and then *Sporadopora* [37] as primitive genera while Cairns [6] suggested *Lepidopora* as the most plesiomorphic genus. All these genera differ from the sketched ancestor for at least one (*Lepidopora* and *Sporadopora*) and two (*Pliobothrus*) character states.

Our results agree with the evolutionary trend proposed by Cairns [7]. Indeed, this author indicated the calcification as the first significant step of the stylasterid evolution and hypothesised the concurrent formation of the gastrostyle. Moreover, he suggested the evolution from polyps randomly arranged to cyclosystem that in the last evolutionary step is protected by a lid. In addition, Cairns considered the lack of dactylostyles as a plesiomorphic state. However, he placed the introduction of double-chambered gastropore tube at the final steps of the evolutionary trend but the results of our analysis are uncertain about this character.

The presence of gastropores with gastrostyle was also noticed in the fossil genus *Axopora* but it lacks dactylopores and ampullae [38]. Cairns [7] has raised the evident contradiction of the occurrence of the earliest known stylasterids (Paleocene) before the earliest known axoporids (Eocene to Oligocene). This contradiction is also evident in the molecular chronogram provided by Lindner et al. [8] where the stylasterid ancestor (that we indirectly know to be provided with dactylopores) is placed at 85 mya. Cairns [7] has hypothesised a solution of this evolutionary problem by the discovery of *Subaxopora* from Late Jurassic. However, the available description of *Subaxopora* do not report presence of dactylopores and ampullae [39].

The present investigation is the first attempt to integrate the molecular and morphological data to better clarify the evolutionary trend of the Stylasteridae. Similarly to the results obtained by Cairns [6], our results indicate that many morphological characters are very plastic and subjected to parallel evolution. These characters are currently used for the stylasterid taxonomy at both generic and specific levels but, given our results, it is clear that they might not reflect the real phylogenetic relationships between the taxa and should be used with caution.

Our analysis allows us to sketch a plausible identikit of the Stylasteridae ancestor that is however not conclusive due to different reasons. Firstly, the available methods to infer the ancestral states of characters have practical and theoretical limits, and as a consequence there is an intrinsic level of uncertainty in this type of reconstruction (e.g. [40]). Secondly and more specifically focused on Stylasteridae, available data set for phylogenetic purposes are still limited, and even the broadest molecular alignment produced [2] so far is based on limited number of genes that are in several cases missing. Thus, as already suggested by Cairns [1], further molecular and morphological analyses are needed to obtain more conclusive results. The implementation of the molecular analysis with other genera, species and new genes may provide a more complete evolutionary scenario and improve the confidence of the ancestor identikit.

## Supporting Information

S1 Fig Neighbor-net computed for DNA.105T set. The neighbor-net shows the conflicting splits (conflicting phylogenetic signals) occurring among the taxa. The neighbor-net was computed with the SplitsTree program, by applying the uncorrected P method.
(PDF)

S2 Fig Neighbor-net computed for DNA.92T set. The neighbor-net shows the conflicting splits (conflicting phylogenetic signals) occurring among the taxa. The neighbor-net was computed with the SplitsTree program, by applying the uncorrected P method.
(PDF)

S3 Fig Neighbor-net computed for MOR.92T set. The neighbor-net shows the conflicting splits (conflicting phylogenetic signals) occurring among the taxa. The neighbor-net was computed with the SplitsTree program, by applying the uncorrected P method.
(PDF)

S4 Fig Maximum likelihood tree obtained from the analysis of DNA.92T data set. The ML tree (-ln = 23438.217) was computed with IQ-TREE program. The scale bar represents 0.02 substitutions per position. Black numbers represent bootstrap values (>50%) expressed in percent, while red numbers refer to Bayesian Inference posterior probabilities.
(PDF)

S5 Fig The LIN cladogram. The LIN cladogram obtained from the ML tree originally published by Lindner et al. (2014), by removing the branches connecting taxa non included in DNA.92T and TOT.92T sets.
(PDF)

S6 Fig Evolution of the characters 1 and 2 mapped on the reference tree obtained from the analysis of DNA.92T data set. The reference cladogram was obtained from the maximum likelihood tree (see S4 Fig), produced in the analysis performed on the DNA.92T data set, by collapsing the nodes which had bootstrap support lower than 50%. A) Character 1, calcareous skeleton. (B) Character 2, coenosteal texture. I, II, and III, major clades cited in the text. The state of the analysed character is represented by a coloured pie, placed at each internal/terminal node of the tree. An enlarged multi-coloured pie is used when multiple states of a character occur at a specific node. In this latter case the size of each slice is proportional to the probability of occurrence of the state.
(PDF)

S7 Fig Evolution of the characters 3a and 3b mapped on the reference tree obtained from the analysis of DNA.92T data set. The reference cladogram was obtained from the maximum likelihood tree (see S4 Fig), produced in the analysis performed on the DNA.92T data set, by collapsing the nodes which had bootstrap support lower than 50%. (A) Character 3a, arrangement of the polyps (three states). (B) Character 3b, arrangements of the polyps (six states). I, II, and III, major clades cited in the text. The state of the analysed character is represented by a coloured pie, placed at each internal/terminal node of the tree. An enlarged multi-coloured pie is used when multiple states of a character occur at a specific node. In this latter case the size of each slice is proportional to the probability of occurrence of the state.
(PDF)

S8 Fig Evolution of the characters 4 and 5 mapped on the reference tree obtained from the analysis of DNA.92T data set. The reference cladogram was obtained from the maximum likelihood tree (see S4 Fig), produced in the analysis performed on the DNA.92T data set, by collapsing the nodes which had bootstrap support lower than 50%. (A) Character 4, absence/presence of cyclosystems. (B) Character 5, arrangement of the cyclosystems (not in pseudocyclosystem form). I, II, and III, major clades cited in the text. The state of the analysed character is represented by a coloured pie, placed at each internal/terminal node of the tree. An enlarged multi-coloured pie is used when multiple states of a character occur at a specific node. In this latter case the size of each slice is proportional to the probability of occurrence of the state.
(PDF)

S9 Fig Evolution of the characters 6 and 7 mapped on the reference tree obtained from the analysis of DNA.92T data set. The reference cladogram was obtained from the maximum likelihood tree (see S4 Fig), produced in the analysis performed on the DNA.92T data set, by collapsing the nodes which had bootstrap support lower than 50%. (A) Character 6, gastropore (gastrostyle presence). (B) Character 7, gastropore (shape of the tube). I, II, and III, major clades cited in the text. The state of the analysed character is represented by a coloured pie, placed at each internal/terminal node of the tree. An enlarged multi-coloured pie is used when multiple states of a character occur at a specific node. In this latter case the size of each slice is proportional to the probability of occurrence of the state.
(PDF)

S10 Fig Evolution of the characters 8 and 9 mapped on the reference tree obtained from the analysis of DNA.92T data set. The reference cladogram was obtained from the maximum likelihood tree (see S4 Fig), produced in the analysis performed on the DNA.92T data set, by collapsing the nodes which had bootstrap support lower than 50%. (A) Character 8, gastropore (lip presence). (B) Character 9, dactylopores (dactylostyle presence). I, II, and III, major clades cited in the text. The state of the analysed character is represented by a coloured pie, placed at each internal/terminal node of the tree. An enlarged multi-coloured pie is used when multiple states of a character occur at a specific node. In this latter case the size of each slice is proportional to the probability of occurrence of the state.
(PDF)

S11 Fig Evolution of the character 10 mapped on the reference tree obtained from the analysis of DNA.92T data set. The reference cladogram was obtained from the maximum likelihood tree (see S4 Fig), produced in the analysis performed on the DNA.92T data set, by collapsing the nodes which had bootstrap support lower than 50%. Character 10, dactylopores (spine presence). I, II, and III, major clades cited in the text. The state of the analysed character is represented by a coloured pie, placed at each internal/terminal node of the tree. An enlarged multi-coloured pie is used when multiple states of a character occur at a specific node. In this latter case the size of each slice is proportional to the probability of occurrence of the state.
(PDF)

S1 Table Morphological matrix. Morphological matrix of the species used in the present analysis; outgroups are shaded in grey.
(PDF)

S2 Table Taxonomic information. List (in alphabetic order) of all the cited genera and species with taxonomic information and related references.
(PDF)

S3 Table Accession numbers in GenBank of gene sequences included in DNA.92T data set. (PDF)
