# Supplementary material for: Integration of Morphological Data into Molecular Phylogenetic Analysis: Toward the Identikit of the Stylasterid Ancestor
Source: PLoS One. 2016 Aug 18;11(8):e0161423. doi: 10.1371/journal.pone.0161423 (PMC4990279; doi:10.1371/journal.pone.0161423)
Supplement: S1 Fig — The neighbor-net shows the conflicting splits (conflicting phylogenetic signals) occurring among the taxa. The neighbor-net was computed with the SplitsTree program, by applying the uncorrected P method. (PDF) [file pone.0161423.s001.pdf]

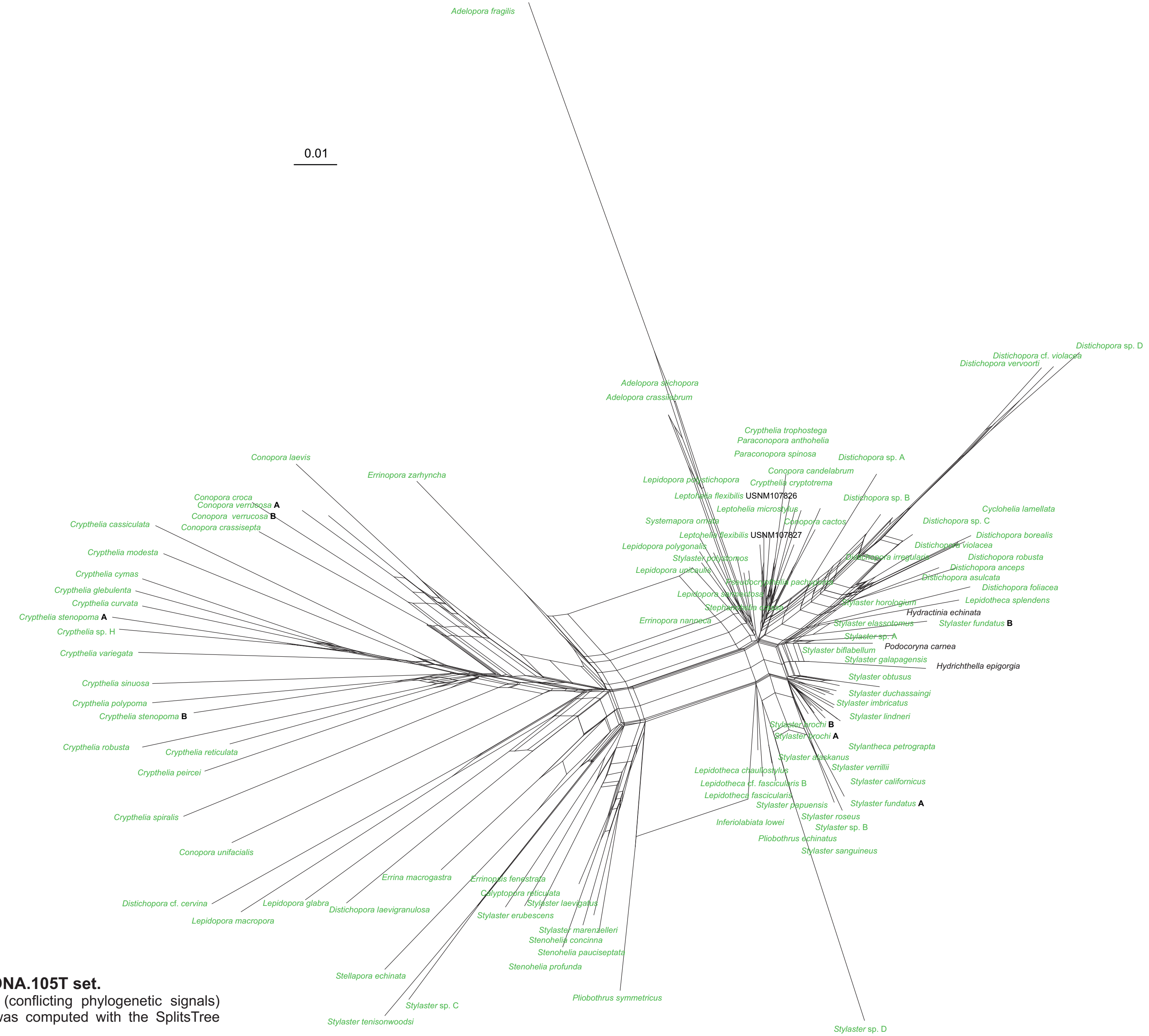

Figure S1. Neighbor-net computed for DNA.105T set.
The neighbor-net shows the conflicting splits (conflicting phylogenetic signals) occurring among the taxa. The neighbor-net was computed with the SplitsTree program, by applying the uncorrected p-distance.
