# Supplementary material for: Integration of Morphological Data into Molecular Phylogenetic Analysis: Toward the Identikit of the Stylasterid Ancestor
Source: PLoS One. 2016 Aug 18;11(8):e0161423. doi: 10.1371/journal.pone.0161423 (PMC4990279; doi:10.1371/journal.pone.0161423)
Supplement: S4 Fig — The ML tree (-ln = 23438.217) was computed with IQ-TREE program. The scale bar represents 0.02 substitutions per position. Black numbers represent bootstrap values (>50%) expressed in percent, while red numbers refer to Bayesian Inference posterior probabilities. (PDF) [file pone.0161423.s004.pdf]

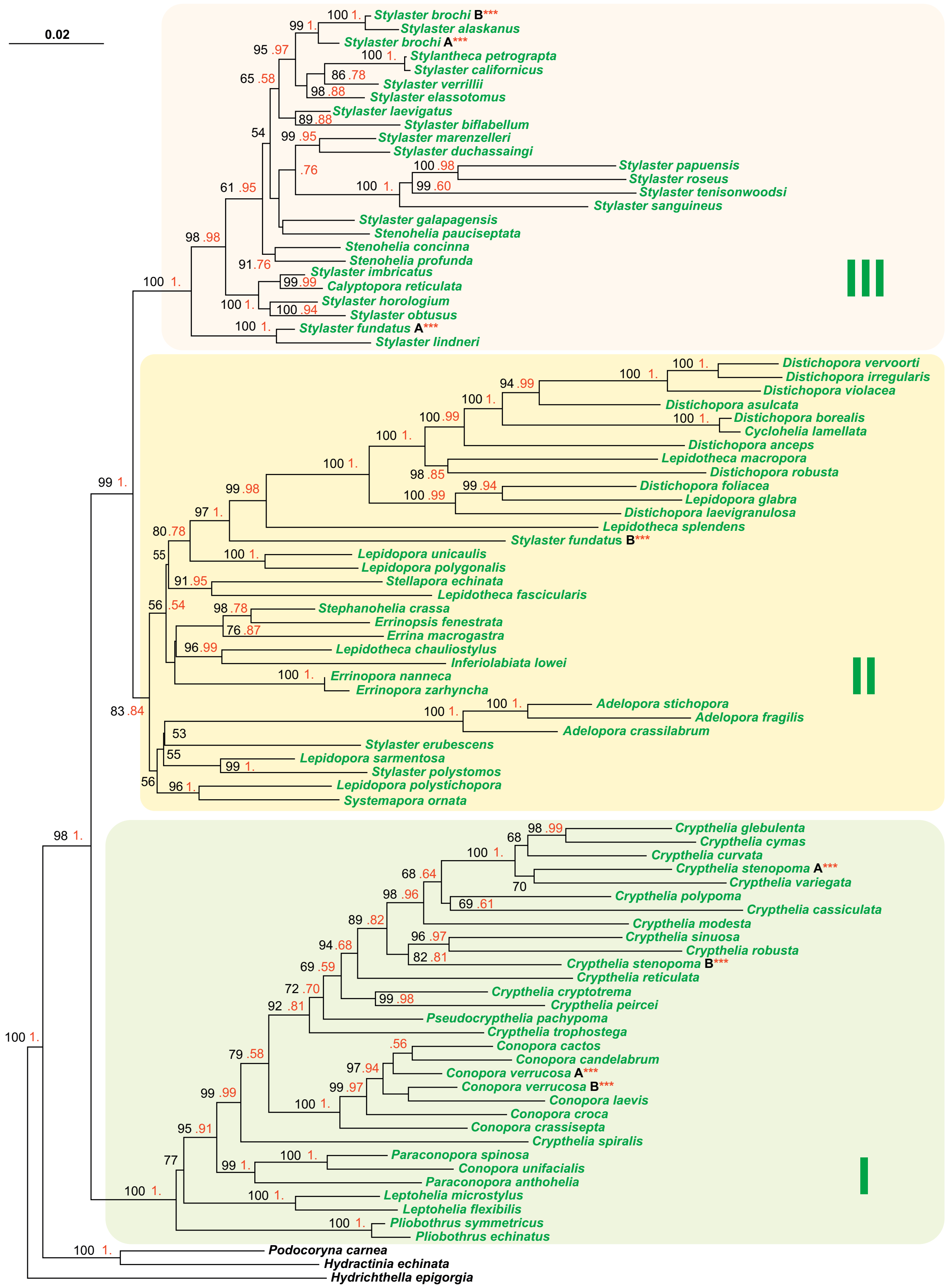

Figure S4. Maximum likelihood tree obtained from the analysis of DNA.92T data set.
The ML tree (-ln = -23438.217) was computed with IQ-TREE program. The scale bar represents 0.02 substitutions per position. Black numbers represent bootstrap values (>50%) expressed in percent, while red numbers refer to Bayesian Inference posterior probabilities.
