# Supplementary material for: Integration of Morphological Data into Molecular Phylogenetic Analysis: Toward the Identikit of the Stylasterid Ancestor
Source: PLoS One. 2016 Aug 18;11(8):e0161423. doi: 10.1371/journal.pone.0161423 (PMC4990279; doi:10.1371/journal.pone.0161423)
Supplement: S5 Fig — The LIN cladogram obtained from the ML tree originally published by Lindner et al. (2014), by removing the branches connecting taxa non included in DNA.92T and TOT.92T sets. (PDF) [file pone.0161423.s005.pdf]

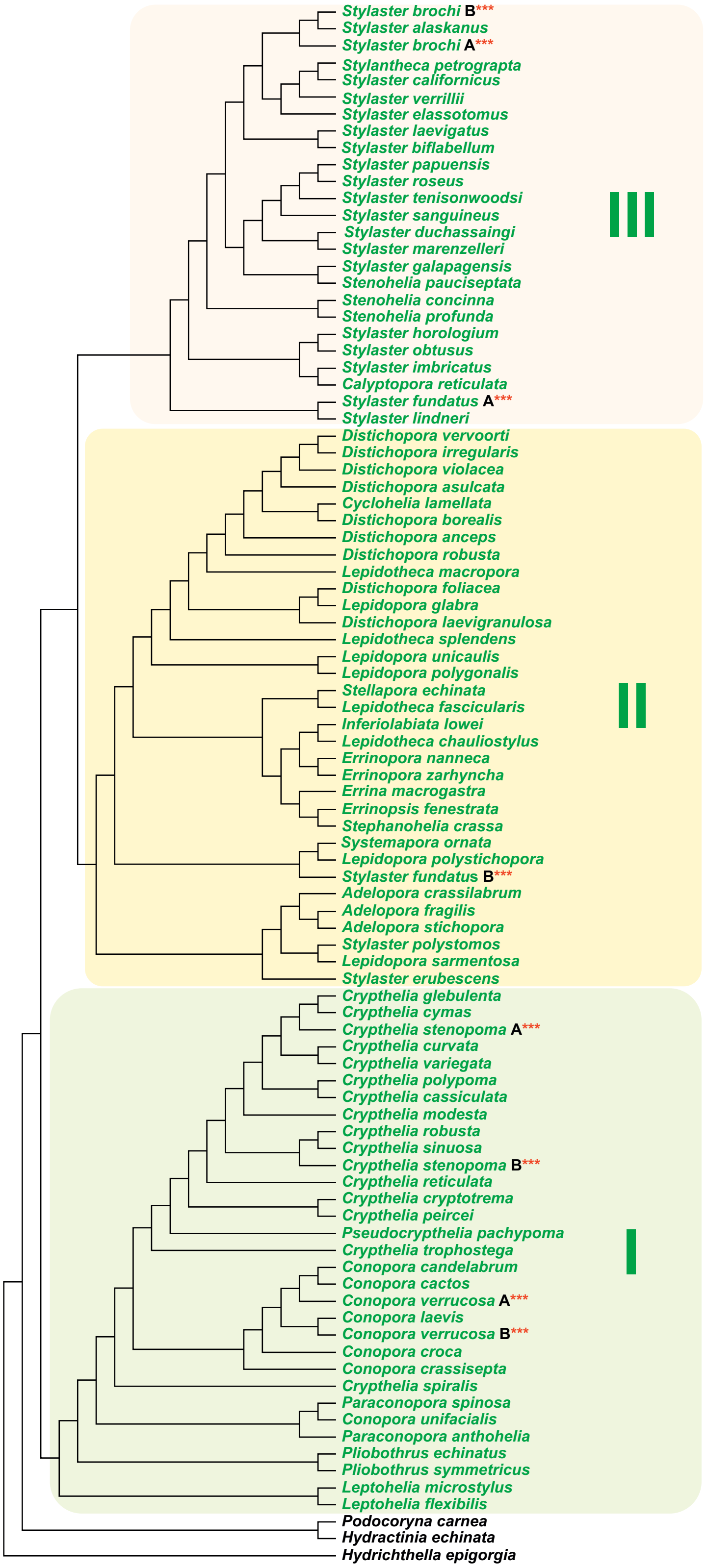

Figure S5. The LIN cladogram.

The LIN cladogram obtained from the ML tree originally published by Lindner et al. [2014], by removing the branches connecting taxa non included in DNA.92T and TOT.92T sets.

Lindner A., Cairns SD, Zibrowius H. *Leptohelia flexibilis* gen. nov. et sp. nov., a remarkable deep-sea stylasterid (Cnidaria: Hydrozoa: Stylasteridae) from the southwest Pacific. Zootaxa 2014; 3900: 581-591.
