# Supplementary material for: Integration of Morphological Data into Molecular Phylogenetic Analysis: Toward the Identikit of the Stylasterid Ancestor
Source: PLoS One. 2016 Aug 18;11(8):e0161423. doi: 10.1371/journal.pone.0161423 (PMC4990279; doi:10.1371/journal.pone.0161423)
Supplement: S7 Fig — The reference cladogram was obtained from the maximum likelihood tree (see S4 Fig), produced in the analysis performed on the DNA.92T data set, by collapsing the nodes which had bootstrap support lower than 50%. (A) Character 3a, arrangement of the polyps (three states). (B) Character 3b, arrangements of the polyps (six states). I, II, and III, major clades cited in the text. The state of the analysed character is represented by a coloured pie, placed at each internal/terminal node of the tree. An enlarged multi-coloured pie is used when multiple states of a character occur at a specific node. In this latter case the size of each slice is proportional to the probability of occurrence of the state. (PDF) [file pone.0161423.s007.pdf]

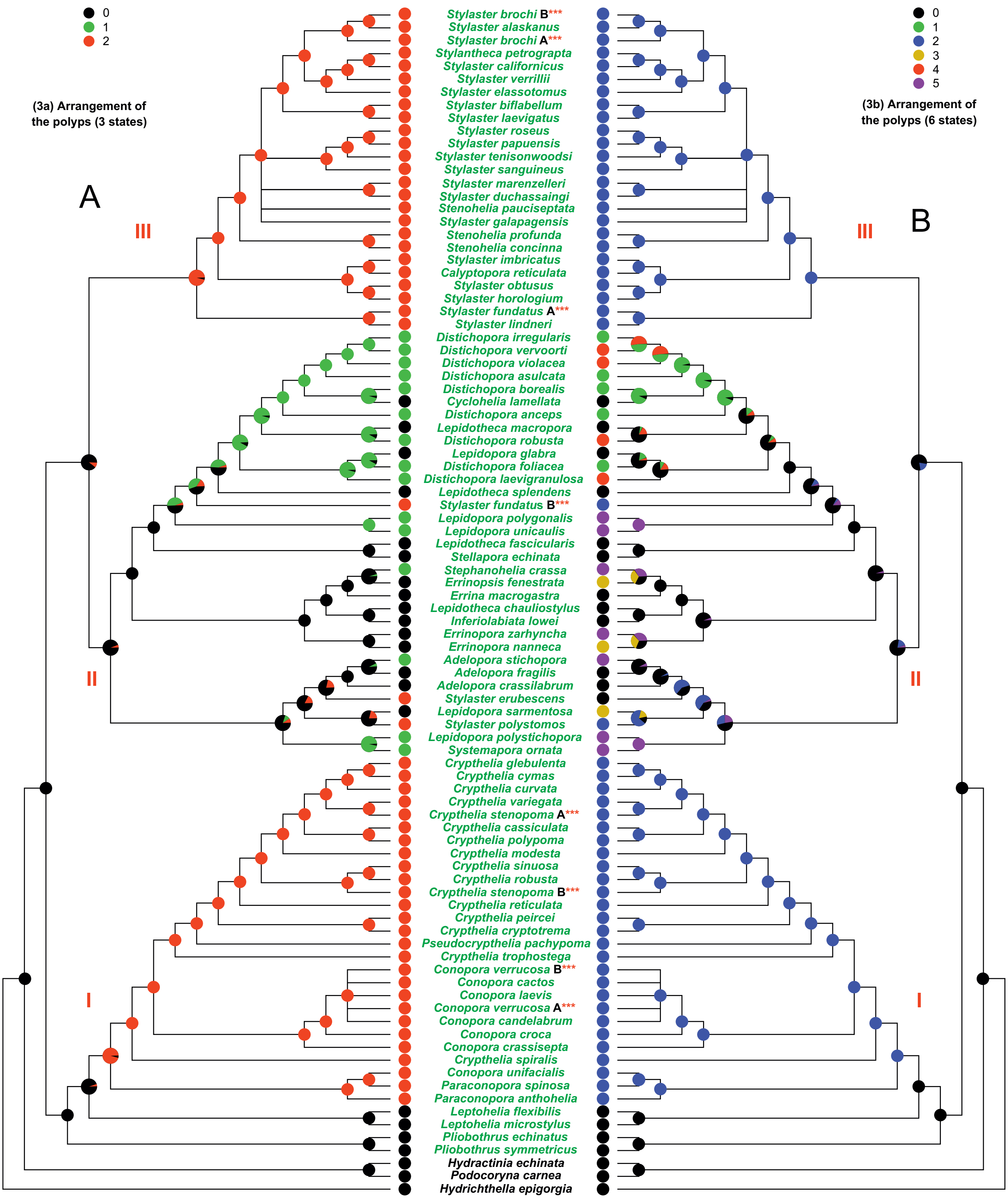

Figure S7. Evolution of the characters 3a and 3b mapped on the reference tree obtained from the analysis of DNA.92T data set.
The reference cladogram was obtained from the maximum likelihood tree (see Figure S4), produced in the analysis performed on the DNA.92T data set, by collapsing the nodes which had bootstrap support lower than 50%.
(A) Character 3a, arrangement of the polyps (three states). (B) Character 3b, arrangements of the polyps (six states). I, II, and III, major clades cited in the text. The state of the analysed character is represented by a coloured pie, placed at each internal/terminal node of the tree. An enlarged multi-coloured pie is used when multiple states of a character occur at a specific node. In this latter case the size of each slice is proportional to the probability of occurrence of the state.
