# Supplementary material for: Integration of Morphological Data into Molecular Phylogenetic Analysis: Toward the Identikit of the Stylasterid Ancestor
Source: PLoS One. 2016 Aug 18;11(8):e0161423. doi: 10.1371/journal.pone.0161423 (PMC4990279; doi:10.1371/journal.pone.0161423)
Supplement: S1 Table — Morphological matrix of the species used in the present analysis; outgroups are shaded in grey. (PDF) [file pone.0161423.s012.pdf]

S1 Table. Morphological matrix. Morphological matrix of the species used in the present analysis; outgroups are shaded in grey.

**Species**	**Characters**										
	**1**	**2**	**3a**	**3b**	**4**	**5**	**6**	**7**	**8**	**9**	**10**
Hydrichthella epigorgia	0	0	0	0	0	0	0	0	0	0	0
Podocoryna carnea	0	0	0	0	0	0	0	0	0	0	0
Hydractinia echinata	0	0	0	0	0	0	0	0	0	0	0
Distichopora vervoorti	1	1	1	4	2	0	1	1	2	2	2
Distichopora irregularis	1	1	1	1	0	0	1	1	2	2	2
Distichopora violacea	1	1	1	4	2	0	1	1	2	2	2
Distichopora asulcata	1	1	1	1	0	0	1	1	2	2	1
Cyclohelia lamellata	1	1	0	0	0	0	1	1	2	2	1
Distichopora borealis	1	1	1	1	0	0	1	1	2	2	1
Distichopora anceps	1	1	1	1	0	0	1	1	2	2	2
Distichopora robusta	1	1	1	4	2	0	1	1	2	2	1
Lepidotheca macropora	1	2	0	0	0	0	1	1	2	2	1
Distichopora foliacea	1	1	1	1	0	0	1	1	2	2	1
Lepidopora glabra	1	1	0	0	0	0	1	1	1	2	1
Distichopora laevigranulosa	1	1	1	4	2	0	1	1	2	2	1
Lepidopora unicaulis	1	3	1	5	0	0	1	1	2	2	1
Inferiolabiata loweii	1	3	0	0	0	0	1	1	2	1	1
Lepidotheca chauliostylus	1	3	0	0	0	0	1	1	1	2	1
Errinopora nanneca	1	1	0	3	2	0	1	1	2	1	1
Errinopora zarhyncha	1	1	0	5	0	0	1	1	2	1	1
Errinopsis fenestrata	1	1	0	3	2	0	1	1	1	2	1
Errina macrogastra	1	2	0	0	0	0	1	1	1	2	1
Stellapora echinata	1	1	0	0	0	0	1	1	2	2	1
Systemapora ornata	1	2	1	5	0	0	1	1	1	2	1
Adelopora fragilis	1	2	0	0	0	0	2	1	1	2	1
Adelopora crassilabrum	1	1	0	0	0	0	2	1	1	2	1
Stylaster erubescens	1	1	2	2	1	2	1	1	2	1	2
Stylaster papuensis	1	?	2	2	1	3	1	1	2	1	2
Stylaster roseus	1	3	2	2	1	2	1	1	2	1	2
Stylaster tenisonwoodsi	1	1	2	2	1	3	1	1	2	1	2
Stylaster sanguineus	1	?	2	2	1	2	1	1	2	?	2
Stylaster duchassaingi	1	2	2	2	1	3	1	1	2	1	2
Stylaster marenzelleri	1	3	2	2	1	2	1	1	2	1	2
Stylaster galapagensis	1	1	2	2	1	3	1	1	2	1	2
Stenohelia pauciseptata	1	3	2	2	1	4	1	1	2	1	2
Stylaster californicus	1	1	2	2	1	1	1	1	2	1	2
Stylantheca petrograpta	1	1	2	2	1	1	1	1	2	1	2
Stylaster verrillii	1	1	2	2	1	1	1	1	2	1	2
Stylaster elassotomus	1	1	2	2	1	3	1	1	2	1	2
Stylaster alaskanus	1	1	2	2	1	3	1	1	2	1	2
*Stylaster brochi*_A	1	1	2	2	1	1	1	1	2	1	2
*Stylaster brochi*_B	1	1	2	2	1	1	1	1	2	1	2

Stylaster laevigatus	1	3	2	2	1	2	1	1	1	1	2
Stenohelia concinna	1	2	2	2	1	4	1	1	2	1	2
Stenohelia profunda	1	3	2	2	1	4	1	1	2	1	2
Stylaster horologium	1	3	2	2	1	3	1	1	2	1	2
Stylaster imbricatus	1	3	2	2	1	3	1	1	2	1	2
Calyptopora reticulata	1	3	2	2	1	5	1	1	1	1	2
*Stylaster fundatus*_A	1	1	2	2	1	3	1	1	2	1	2
Crypthelia cymas	1	2	2	2	1	4	2	2	2	2	2
Crypthelia glebulenta	1	2	2	2	1	4	2	2	2	2	2
Crypthelia curvata	1	2	2	2	1	4	2	2	2	2	2
Crypthelia polypoma	1	2	2	2	1	5	2	2	2	2	2
Crypthelia robusta	1	2	2	2	1	5	2	2	2	2	2
Crypthelia cryptotrema	1	2	2	2	1	5	2	2	2	2	2
Crypthelia peircei	1	2	2	2	1	4	2	2	2	2	2
Pseudocrypthelia pachypoma	1	2	2	2	1	4	1	2	2	2	2
Crypthelia trophostega	1	2	2	2	1	5	2	2	2	2	2
Conopora candelabrum	1	3	2	2	1	3	2	2	2	2	2
*Conopora verrucosa*_A	1	2	2	2	1	3	2	2	2	2	2
Conopora laevis	1	2	2	2	1	3	2	2	2	2	2
Pliobothrus symmetricus	1	2	0	0	0	0	2	2	2	2	1
Pliobothrus echinatus	1	2	0	0	0	0	2	2	2	2	1
Leptohelia microstylus	1	1	0	0	0	0	1	2	1	2	1
Paraconopora anthohelia	1	3	2	2	1	5	1	1	2	2	2
Leptohelia flexibilis	1	1	0	0	0	0	1	2	1	2	1
Paraconopora spinosa	1	2	2	2	1	4	1	1	2	2	2
Conopora unifacialis	1	2	2	2	1	4	2	2	2	2	2
Conopora cactos	1	2	2	2	1	1	2	2	2	2	2
*Conopora verrucosa*_B	1	2	2	2	1	3	2	2	2	2	2
Conopora croca	1	2	2	2	1	3	2	2	2	2	2
Conopora crassisepta	1	2	2	2	1	3	2	2	2	2	2
Crypthelia spiralis	1	2	2	2	1	3	2	2	2	2	2
Crypthelia cassiculata	1	2	2	2	1	4	2	2	2	2	2
Crypthelia modesta	1	2	2	2	1	4	2	2	2	2	2
*Crypthelia stenopoma*_A	1	2	2	2	1	4	2	2	2	2	2
*Crypthelia stenopoma*_B	1	2	2	2	1	4	2	2	2	2	2
Crypthelia reticulata	1	3	2	2	1	5	2	2	2	2	2
Crypthelia variegata	1	2	2	2	1	5	2	2	2	2	2
Crypthelia sinuosa	1	2	2	2	1	5	2	2	2	2	2
Stylaster polystomos	1	2	2	2	1	3	1	1	2	1	2
Stylaster lindneri	1	3	2	2	1	3	1	1	2	1	2
Stylaster obtusus	1	3	2	2	1	2	1	1	2	1	2
Stylaster biflabellum	1	2	2	2	1	3	1	1	2	1	2
*Stylaster fundatus*_B	1	1	2	2	1	3	1	1	2	1	2
Lepidopora sarmentosa	1	1	0	3	2	0	1	1	2	2	1
Lepidopora polystichopora	1	3	1	5	0	0	1	1	1	2	1

Lepidopora polygonalis	1	3	1	5	0	0	1	1	1	2	1
Lepidotheca fascicularis	1	2	0	0	0	0	1	1	1	2	1
Lepidotheca splendens	1	3	0	0	0	0	1	1	2	2	1
Adelopora stichopora	1	2	1	5	0	0	2	1	1	2	1
Stephanohelia crassa	1	2	1	5	2	0	1	1	2	2	1
