# Supplementary material for: Integration of Morphological Data into Molecular Phylogenetic Analysis: Toward the Identikit of the Stylasterid Ancestor
Source: PLoS One. 2016 Aug 18;11(8):e0161423. doi: 10.1371/journal.pone.0161423 (PMC4990279; doi:10.1371/journal.pone.0161423)
Supplement: S2 Table — List (in alphabetic order) of all the cited genera and species with taxonomic information and related references. (PDF) [file pone.0161423.s013.pdf]

S2 Table. Taxonomic information. List (in alphabetic order) of all the cited genera and species with taxonomic information and related references.

**Genus**	**Species**
Axopora Milne Edwards & Haime, 1850 [1]	
Adelopora Cairns, 1982 [2]	
	Adelopora crassilabrum Cairns, 1991 [3]
	Adelopora fragilis Cairns, 1991 [3]
	Adelopora stichopora Cairns, 2015 [4]
Calyptopora Boschma, 1968 [5]	
	Calyptopora reticulata Boschma, 1968 [5]
Cheiloporidion Cairns, 1983 [6]	
	Cheiloporidion pulvinatum Cairns, 1983 [6]
Conopora Moseley, 1879 [7]	
	Conopora cactos Cairns, 2015 [4]
	Conopora candelabrum Cairns, 1991 [3]
	Conopora crassisepta Cairns, 2015 [4]
	Conopora croca Cairns, 2015 [4]
	Conopora laevis (Studer, 1878) [8]
	Conopora verrucosa (Studer, 1878) [8]
	Conopora unifacialis Cairns, 1991 [3]
Crypthelia Milne Edwards & Haime, 1849 [9]	
	Crypthelia cassiculata Cairns, 2015 [4]
	Crypthelia cryptotrema Zibrowius, 1981 [10]
	Crypthelia curvata Cairns, 1991 [3]
	Crypthelia cymas Cairns, 1986 [11]
	Crypthelia glebulenta Cairns, 1986 [11]
	Crypthelia modesta Cairns, 2015 [4]
	Crypthelia peircei Pourtalès, 1867 [12]
	Crypthelia polypoma Cairns, 1991 [3]
	Crypthelia reticulata Cairns, 2015 [4]
	Crypthelia robusta Cairns, 1991[3]
	Crypthelia sinuosa Cairns, 2015 [4]
	Crypthelia spiralis Cairns, 2015 [4]
	Crypthelia stenopoma (Hickson & England, 1905) [13]
	Crypthelia trophostega Fisher, 1938 [14]
	Crypthelia variegata Cairns, 2015 [4]
Cyclohelia Cairns, 1991 [15]	
	Cyclohelia lamellata Cairns, 1991 [15]
Distichopora Lamarck, 1816 [16]	
	Distichopora anceps Cairns, 1978 [17]
	Distichopora asulcata Cairns, 2005 [18]
	Distichopora borealis Fisher, 1938 [14]
	Distichopora foliacea Pourtalès, 1868 [19]
	Distichopora irregularis Moseley, 1879 [7]
	Distichopora laevigranulosa Cairns, 1986 [11]

	Distichopora robusta Lindner, Cairns & Guzman, 2004 [20]
	Distichopora vervoorti Cairns & Hoeksema, 1998 [21]
	Distichopora violacea (Pallas, 1766) [22]
Errina Gray, 1835 [23]	
	Errina macrogastra Marenzeller, 1904 [24]
Errinopora Fisher, 1938 [14]	
	Errinopora nanneca Fisher, 1938 [14]
	Errinopora zarhyncha Fisher, 1938 [14]
Errinopsis Broch, 1951 [25]	
	Errinopsis fenestrata Cairns, 1983 [6]
Hydractinia Van Beneden, 1844 [26]	
	Hydractinia echinata (Fleming, 1828) [27]
Hydrichthella Stechow, 1909 [28]	
	Hydrichthella epigorgia Stechow, 1909 [28]
Inferiolabiata Broch, 1951 [25]	
	Inferiolabiata loweii (Cairns, 1983) [6]
Lepidopora Pourtalès, 1871 [29]	
	Lepidopora glabra (Pourtalès, 1867) [12]
	Lepidopora polygonalis Cairns, 2015 [4]
	Lepidopora polystichopora Cairns, 1985 [30]
	Lepidopora sarmentosa (Boschma, 1968) [31]
	Lepidopora unicaulis Cairns, 2015 [4]
Lepidotheca Cairns, 1983 [32]	
	Lepidotheca chauliostylus Cairns, 1991 [3]
	Lepidotheca fascicularis Cairns, 1983 [6]
	Lepidotheca macropora Cairns, 1986 [11]
	Lepidotheca splendens Cairns, 2015 [4]
Leptohelia Lindner, Cairns & Zibrowius, 2014 [33]	
	Leptohelia flexibilis Lindner, Cairns & Zibrowius, 2014 [33]
	Leptohelia microstylus (Cairns, 1991) [3]
Paraconopora Cairns, 2015 [4]	
	Paraconopora anthohelia (Cairns, 1991) [3]
	Paraconopora spinosa Cairns, 2015 [4]
Pliobothrus Pourtalès, 1868 [19]	
	Pliobothrus echinatus Cairns, 1986 [34]
	Pliobothrus symmetricus Pourtalès, 1868 [19]
Podocoryna M. Sars, 1846 [35]	
	Podocoryna carnea M. Sars, 1846 [35]
Pseudocrypthelia Cairns, 1983 [36]	
	Pseudocrypthelia pachypoma (Hickson & England, 1905) [13]
Sporadopora Moseley, 1879 [7]	
Stellapora Cairns, 1983 [32]	
	Stellapora echinata (Moseley, 1879) [7]
Stenohelia Kent, 1870 [37]	
	Stenohelia concinna Boschma, 1964 [38]

	Stenohelia pauciseptata Cairns, 1986 [34]
	Stenohelia profunda Moseley, 1881 [39]
Stephanohelia Cairns, 1991 [3]	
	Stephanohelia crassa Cairns, 2015 [4]
Stylantheca Fischer, 1931 [40]	
	Stylantheca petrograpta (Fisher, 1938) [14]
Stylaster Gray, 1831 [41]	
	Stylaster alaskanus Fisher, 1938 [14]
	Stylaster biflabellum Cairns, 2015 [4]
	Stylaster brochi (Fisher, 1938) [14]
	Stylaster brunneus Boschma 1970 [42]
	Stylaster californicus (Verrill, 1866) [43]
	Stylaster duchassaingi Pourtalès, 1867 [12]
	Stylaster elassotomus Fisher, 1938 [14]
	Stylaster erubescens Pourtalès, 1868 [19]
	Stylaster fundatus Cairns, 2015 [4]
	Stylaster galapagensis Cairns, 1986 [11]
	Stylaster horologium Cairns, 1991 [3]
	Stylaster imbricatus Cairns, 1991 [3]
	Stylaster laevigatus Cairns, 1986 [34]
	Stylaster lindneri Cairns, 2015 [4]
	Stylaster marenzelleri Cairns, 1986 [11]
	Stylaster obtusus Cairns, 2015 [4]
	Stylaster papuensis Zibrowius, 1981 [10]
	Stylaster polystomos Cairns, 2015 [4]
	Stylaster roseus (Pallas, 1766) [22]
	Stylaster sanguineus Valenciennes in Milne Edwards & Haime, 1850 [1]
	Stylaster tenisonwoodsi Cairns, 1988 [44]
	Stylaster verrillii (Dall, 1884) [45]
Subaxopora Deng, 1982 [46]	
Systemapora Cairns, 1991 [3]	
	Systemapora ornata Cairns, 1991 [3]

References

1. Milne Edwards H, Haime J. Recherches sur les Polypiers. 5me Mém Monographie des Oculinides. Ann Sc Nat, Zool. 1850; vol. 13.

2. Cairns SD. A new subfamily of operculate stylasterine (Coelenterata; Hydrozoa) from the Subantarctic. J Nat Hist. 1982; 16: 71-81.

3. Cairns SD. The Marine Fauna of New Zealand: Stylasteridae (Cnidaria: Hydroida). Mem NZ Oceanogr Inst. 1991; 98: 1-99.

4. Cairns S D. Stylasteridae (Cnidaria: Hydrozoa: Anthoathecata) of the New Caledonian region. Mém Mus Natl Hist Nat 2015; 207: 1-362.

5. Boschma H. *Calvptopora reticulata* n. g., n. sp., a stylasterine coral from deep water in the New Zealand region. Proc K Ned Akad Wet. 1968; 71: 99-108.

6. Cairns SD. Antarctic and Subantarctic Stylasterina (Coelenterata: Hydrozoa). Antarct Res Ser. 1983; 38: 61-164.

7. Moseley HN. On the structure of the Stylasteridae, a family of the hydroid stony corals. Phil Trans R Soc Lond. 1879; 169: 425-503.

8. Studer T. Ubersicht der Steinkorallen aus der Familie der *Madreporaria aporosa, Eupsammina* and *Turbinarina*, welche auf der Reise S.M.S. Gazelle um die Erde gesammelt wurden. Monatber Kon Preuss Akad Wiss, Berlin. 1878; 625-654

9. Milne Edwards H, Haime J. Mémoire sur les polypiers appartenant à la famille des oculinides, au groupe intermédiaire des Pseudoastréides et à la famille des Fongides. CR Hebd Séances Acad Sci. 1849; 29: 67-73.

10. Zibrowius H. Associations of Hydrocorallia Stylasterina with gall-inhabiting Copepoda Siphonostomatoidea from the south-west Pacific. Part I. On the stylasterine hosts, including two new species, *Stylaster papuensis* and *Crypthelia cryptotrema*. Bijdr Dierk. 1981; 51: 268-281.

11. Cairns SD. Stylasteridae (Hydrozoa: Hydroida) of the Galapagos Islands. Smithson Contr Zool. 1986; 426: 1-42.

12. Pourtalès, LF de. Contributions to the fauna of the Gulf Stream at great depths. Bull Mus Comp Zool Harv. 1867; 1: 103-120.

13. Hickson SJ, England HM. The Stylasterina of the Siboga Expedition. Siboga-Expeditie Monogr VIII. Leiden. 1905.

14. Fisher WK. Hydrocorals of the North Pacific Ocean. Proc US Nat Mus. 1938; 84: 493-554.

15. Cairns SD. *Cyclohelia lamellata*, new genus and species of Stylasteridae (Cnidaria: Hydrozoa) from the Bering Sea. Pacif Sci 1991; 45: 383-388.

16. Lamarck JBPA de M. Histoire naturelle des animaux sans vertèbres, 2. Paris: Verdière. 1816.

17. Cairns SD. *Distichopora* (*Haplomerismos*) *anceps*, a new Stylasterine coral (Coelenterata: Stylasterina) from deep water off the Hawaiian Islands. Micronesica. 1978; 14: 83-87.

18. Cairns SD. Revision of the Hawaiian Stylasteridae (Cnidaria: Hydrozoa: Athecata). Pac Sci. 2005; 59: 439-451.

19. Pourtalès, LF de. Contributions to the fauna of the Gulf Stream at great depths (2 series). Bull Mus Comp Zool Harv. 1868; 1: 121-142.

20. Lindner A, Cairns SD, Guzman M. *Distichopora robusta* sp. nov., the first shallow-water stylasterid (Cnidaria: Hydrozoa: Stylasteridae) from the tropical eastern Pacific. J Mar Biol Ass UK. 2004; 84: 943-947.

21. Cairns SD., Hoeksema BW. *Distichopora vervoorti*, a new shallow-water stylasterid coral (Cnidaria: Hydrozoa: Stylasteridae) from Bali, Indonesia. Zool Verh Leiden. 1998; 323: 311-318.

22. Pallas PS. Elenchus Zoophytorum sistens generum adumbrations generaliores et specierum cognitarum succinctas descriptions cum selectis auctorum synonymis. Hagae-Comitum. 1766.

23. Gray JE. Characters of two new genera of corals, *Errina* and *Anthophora*. Proc Zool Soc Lond.

1835; 1835: 85-86.

24. Marenzeller E Von. Stein- und Hydro-Korallen. Bull Mus Comp Zool. 1904; 43: 75-87

25. Broch H. Slylasteridae (Hydrocorals) from the Southern Seas. "Discovery" Rep. 1951; 26: 33-46.

26. Van Beneden PJ. Sur les genres *Eleutherie* et *Synhydre*. Bull Acad Roy Sci Bell Lettres Bruxelles. 1844; 11: 305-314.

27. Fleming J. A History of British Animals, Exhibiting the Descriptive Characters and Systematical Arrangement of the Genera and Species of Quadrupeds, Birds, Reptiles, Fishes, Mollusca, and Radiata of the United Kingdom; including the Indigenous, Extirpated, and Extinct Kinds, together with Periodical and Occasional Visitants. Edinburgh: Bell and Bradfute; 1828.

28. Stechow E. Hydroidpolypen der japanische Ostküste. I. Teil: Athecata und Plumularidae. Abh bayer Akad Wiss, Suppl Bd. 1909; 1-111

29. Pourtalès, LF de. Deep-sea corals. Illust Cat Mus Comp Zool Harv. 1871; 4: 1-93.

30. Cairns SD. Three new species of Stylasteridae (Coelenterata: Hydrozoa). Proc Biol Soc Wash. 1985; 98: 728-739.

31. Boschma H. *Errina sarmentosa*, a new stylasterine coral from deep water in the New Zealand region. Proc. Kon. Ned. Akad. Wet. 1968; 71: 203-208.

32. Cairns SD. A generic revision of the Stylasterina (Coelenterata: Hydrozoa). Part 1. Description of the genera. Bull Mar Sci. 1983; 33: 427-508.

33. Lindner A., Cairns SD, Zibrowius H. *Leptohelia flexibilis* gen. nov. et sp. nov., a remarkable deep-sea stylasterid (Cnidaria: Hydrozoa: Stylasteridae) from the southwest Pacific. Zootaxa. 2014; 3900: 581-591.

34. Cairns SD. A revision of the Northwest Atlantic Stylasteridae (Coelenterata: Hydrozoa). Smithson Contr Zool 1986; 418: 1-131.

35. Sars M. Fauna littoralis Norvegiae. 1. Heft. I. Ueber die Fortpflanzungsweise einiger Polypen Johann Dahl, Christiania. 1846.

36. Cairns SD. *Pseudocrypthelia*, a new genus of stylasterine coral (Coelenterata: Hydrozoa) from

the Indonesian region. Beaufortia. 1983; 33: 29-35.

37. Kent WS. On a new genus of the Madreporaria or stony corals (*Stenohelia*). J Nat Hist. 1870; 5: 120-123.

38. Boschma H. Further notes on the stylasterine coral *Stenohelia concinna*. Proc Kon Ned Akad Wet. 1964; 61:14-77.

39. Moseley HN. Report on certain hydroid, Alcyonarian and Madreporarian corals procured during the voyage of H. M. S. Challenger in the years 1873–1876. Part 1: On the Hydrocoralline. Rep Sci Res Voyage H M S Challenger, Zoology. 1881; 2:. 1–181, 209-230.

40. Fisher WK. Californian Hydrocorals. J Nat Hist. 1931; 8: 391-399.

41. Gray JE. Description of a new genus (*Stylaster*) of star-bearing corals. Zool Misc. 1831; 1831: 36-37.

42. Boschma H. *Stylaster brunneus*, a new stylasterine coral from New Caledonia. Proc Kon Ned Akad Wet. 1970; 73: 154-158.

43. Verrill AE. Synopsis of the polyps and corals of the North Pacific Exploring Expedition under Commodore C. Ringgold and Captain John Rodgers, U.S.N., from 1853 to 1856; collected by Wm. Stimpson, naturalist to the expedition; with descriptions of some additional new species from the west coast of North America. Part 3: Madreporaria. Comm Essex Inst. 1866; 5:17-50.

44. Cairns SD. New records of Stylasteridae (Cnidaria: Hydrozoa) from Western Australia, including the description of two new species. Rec W Aust Mus. 1988; 14: 105-119.

45. Dall WH. On some Hydrocorallinae from Alaska and California. Proc Biol Soc Wash. 1884; 2: 111–115.

46. Deng Z. Mesozoic Milleporina and tabulatomorphic corals from Xizang. Paleontol Xizang. 1982; 4: 184-188.
