# Supplementary material for: Integration of Morphological Data into Molecular Phylogenetic Analysis: Toward the Identikit of the Stylasterid Ancestor
Source: PLoS One. 2016 Aug 18;11(8):e0161423. doi: 10.1371/journal.pone.0161423 (PMC4990279; doi:10.1371/journal.pone.0161423)
Supplement: S3 Table — (PDF) [file pone.0161423.s014.pdf]

S3 Table. Accession numbers in GenBank of gene sequences included in DNA.92T data set (1/2)

	Taxon	16S	18S	calM
1	*Hydrichthella epigorgia*	EU305478	EU272622	***
2	*Hydractinia echinata*	FJ214556	JQ407378	JQ437282
3	*Podocoryna carnea*	FJ214469	JQ407393	JQ437297
4	*Adelopora crassilabrum*	EU645356	EU272642	EU645421
5	*Adelopora fragilis*	EU645355	***	***
6	*Adelopora stichopora*	EU645354	EU645474	EU645420
7	*Calyptopora reticulata*	EU645297	***	***
8	*Conopora cactos*	EU645271	EU645431	EU645370
9	*Conopora candelabrum*	EU645275	EU645432	EU645371
10	*Conopora crassisepta*	EU645277	***	***
11	*Conopora croca*	EU645276	***	***
12	*Conopora laevis*	EU645272	***	***
13	*Conopora unifacialis*	EU645270	***	***
14	*Conopora verrucosa* **A**	EU645273	***	***
15	*Conopora verrucosa* **B**	EU645274	***	***
16	*Crypthelia cassiculata*	EU645289	***	***
17	*Crypthelia cryptotrema*	EU645281	EU272641	EU645374
18	*Crypthelia curvata*	EU645287	***	***
19	*Crypthelia cymas*	EU645284	***	***
20	*Crypthelia glebulenta*	EU645283	***	***
21	*Crypthelia modesta*	EU645290	***	***
22	*Crypthelia peircei*	EU645282	***	***
23	*Crypthelia polypoma*	EU645291	***	***
24	*Crypthelia reticulata*	EU645292	***	***
25	*Crypthelia robusta*	EU645295	***	***
26	*Crypthelia sinuosa*	EU645294	***	***
27	*Crypthelia spiralis*	EU645279	***	***
28	*Crypthelia stenopoma* **A**	EU645288	***	***
29	*Crypthelia stenopoma* **B**	EU645293	***	***
30	*Crypthelia trophostega*	EU645278	EU645433	EU645372
31	*Crypthelia variegata*	EU645286	***	***
32	*Cyclohelia lamellata*	EU645353	EU645473	EU645419
33	*Distichopora anceps*	EU645341	EU645465	EU645409
34	*Distichopora asulcata*	EU645343	EU645467	EU645411
35	*Distichopora borealis*	EU645342	EU645466	EU645410
36	*Distichopora foliacea*	EU645351	EU645472	***
37	*Distichopora irregularis*	EU645344	EU645468	EU645412
38	*Distichopora laevigranulosa*	EU645352	***	EU645418
39	*Distichopora robusta*	EU645339	EU645464	EU645408
40	*Distichopora vervoorti*	EU645345	EU645345	EU645413
41	*Distichopora violacea*	EU645349	EU645470	EU645417
42	*Errina macrogastra*	EU645360	***	***
43	*Errinopora nanneca*	EU645358	EU645475	EU645423
44	*Errinopora zarhyncha*	EU645359	***	***
45	*Errinopsis fenestrata*	EU645357	***	EU645422
46	*Inferiolabiata loweii*	EU645361	EU645476	EU645424
47	*Lepidopora glabra*	EU645328	***	***

***, sequence not existing; 16S, 16S partial gene; 18S, 18S partial gene; calM, calmodulin partial gene.

S3 Table. Accession numbers in GenBank of gene sequences included in DNA.92T data set (2/2)

	Taxon	16S	18S	calM
48	*Lepidopora polygonalis*	EU645333	EU645459	EU645403
49	*Lepidopora polystichopora*	EU645331	EU645457	EU645401
50	*Lepidopora sarmentosa*	EU645330	EU645456	EU645400
51	*Lepidopora unicaulis*	EU645332	EU645458	EU645402
52	*Lepidotheca chauliostylus*	EU645362	EU645477	EU645425
53	*Lepidotheca fascicularis*	EU645334	EU645460	EU645404
54	*Lepidotheca macropora*	EU645336	***	***
55	*Lepidotheca splendens*	EU645337	EU645462	EU645406
56	*Leptohelia flexibilis*	KM886597	KM886599	KM886601
57	*Leptohelia microstylus*	EU645329	EU272644	EU645399
58	*Paraconopora anthohelia*	EU645268	EU645429	EU645368
59	*Paraconopora spinosa*	EU645269	EU645430	EU645369
60	*Pliobothrus echinatus*	EU645266	EU645428	EU645366
61	*Pliobothrus symmetricus*	EU645267	***	EU645367
62	*Pseudocrypthelia pachypoma*	EU645280	EU272643	EU645373
63	*Stellapora echinata*	EU645363	***	***
64	*Stenohelia concinna*	EU645324	***	EU645396
65	*Stenohelia pauciseptata*	EU645325	***	EU645397
66	*Stenohelia profunda*	EU645326	***	***
67	*Stephanohelia crassa*	EU645364	EU645478	EU645426
68	*Stylantheca petrograpta*	EU645327	EU645455	EU645398
69	*Stylaster alaskanus*	EU645308	EU645444	EU645386
70	*Stylaster biflabellum*	EU645307	EU645443	EU645385
71	*Stylaster brochi* **A**	EU645309	EU645445	EU645387
72	*Stylaster brochi* **B**	EU645306	EU645442	EU645384
73	*Stylaster californicus*	EU645314	EU645449	EU645392
74	*Stylaster duchassaingi*	EU645303	EU645440	EU645381
75	*Stylaster elassotomus*	EU645310	EU645446	EU645388
76	*Stylaster erubescens*	EU645322	***	EU645394
77	*Stylaster fundatus* **A**	EU645296	EU645434	EU645375
78	*Stylaster fundatus* **B**	EU645323	EU645454	EU645395
79	*Stylaster galapagensis*	EU645305	EU645441	EU645383
80	*Stylaster horologium*	EU645301	EU645438	EU645379
81	*Stylaster imbricatus*	EU645313	EU645448	EU645391
82	*Stylaster laevigatus*	EU645312	***	EU645390
83	*Stylaster lindneri*	EU645299	EU645436	EU645377
84	*Stylaster marenzelleri*	EU645304	***	EU645382
85	*Stylaster obtusus*	EU645300	EU645437	EU645378
86	*Stylaster papuensis*	EU645316	EU645451	***
87	*Stylaster polystomos*	EU645298	EU645435	EU645376
88	*Stylaster roseus*	EU645315	EU645450	EU645393
89	*Stylaster sanguineus*	EU645321	EU645453	***
90	*Stylaster tenisonwoodsi*	EU645319	***	***
91	*Stylaster verrillii*	EU645311	EU645447	EU645389
92	*Systemapora ornata*	EU645365	EU645479	EU645427

***, sequence not existing; 16S, 16S partial gene; 18S, 18S partial gene; calM, calmodulin partial gene.

S3 Table. summary

	16S	18S	calM
Sequence not existing	0/92	37/92	33/92
